# Poor reporting quality of observational clinical studies comparing treatments of COVID-19 – a retrospective cross-sectional study

**DOI:** 10.1186/s12874-021-01501-9

**Published:** 2022-01-20

**Authors:** Sebastian Ziemann, Irina Paetzolt, Linda Grüßer, Mark Coburn, Rolf Rossaint, Ana Kowark

**Affiliations:** 1 Department of Anaesthesiology, Medical Faculty, RWTH Aachen University, Aachen, Germany; 2 Department of Anaesthesiology and Intensive Care Medicine, University Hospital Bonn, Bonn, Germany

**Keywords:** Reporting quality, COVID-19, Observational studies, STROBE statement

## Abstract

**Background:**

During the COVID-19 pandemic, the scientific world is in urgent need for new evidence on the treatment of COVID patients. The reporting quality is crucial for transparent scientific publication. Concerns of data integrity, methodology and transparency were raised. Here, we assessed the adherence of observational studies comparing treatments of COVID 19 to the STROBE checklist in 2020.

**Methods:**

Design: We performed a retrospective, cross-sectional study. Setting: We conducted a systematic literature search in the Medline database. This study was performed at the RWTH Aachen University Hospital, Department of Anaesthesiology Participants: We extracted all observational studies on the treatment of COVID-19 patients from the year 2020. Main outcome measures: The adherence of each publication to the STROBE checklist items was analysed. The journals’ impact factor (IF), the country of origin, the kind of investigated treatment and the month of publication were assessed.

**Results:**

We analysed 147 observational studies and found a mean adherence of 45.6% to the STROBE checklist items. The percentage adherence per publication correlated significantly with the journals’ IF (point estimate for the difference between 1^st^ and 4^th^ quartile 11.07%, 95% CI 5.12 to 17.02, *p* < 0.001). U.S. American authors gained significantly higher adherence to the checklist than Chinese authors, mean difference 9.10% (SD 2.85%, *p* = 0.023).

**Conclusions:**

We conclude a poor reporting quality of observational studies on the treatment of COVID-19 throughout the year 2020. A considerable improvement is mandatory.

**Supplementary Information:**

The online version contains supplementary material available at 10.1186/s12874-021-01501-9.

## Background

Beginning in late 2019, the entire world was confronted with a global, rapidly spreading pandemic, caused by the *severe acute respiratory syndrome coronavirus2* (SARS-CoV-2). Starting in the region of Wuhan, China, patients suffering from coronavirus disease 2019 (COVID-19) flooded the hospitals worldwide. The huge number of patients, especially those who required treatment on intensive care units and even ventilation, surpassed the capacity limits of several healthcare systems. Health care professionals all over the world were seeking for reliable information on how best to treat COVID-19 patients. Researchers worldwide were under enormous time pressure to provide evidence for the best therapeutic strategies of this unknown disease. Interventional studies, especially randomised controlled trials (RCTs), are considered to be the “gold standard” for gaining evidence on the most effective treatment options for health care professionals [1]. However, the conduct of interventional studies is preceded by extensive regulatory burdens and data acquisition is long-lasting due to the mandatory prospective study design. Thus, in the rapidly evolving situation in the first year of the pandemic, evidence from RTCs on treatment options of COVID-19 was scarce and most evidence was provided within the framework of observational studies. Manuscripts were written, submitted, reviewed and published under particular time constraints in order to provide health care professionals with new knowledge as fast as possible. Also, healthcare professionals searching for the best therapeutic options for COVID-19 patients were faced to enormous time constraints in consideration of the extraordinary circumstances. Leading medical journals reported more than twice as many manuscript submissions in the first half of 2020 than in the pre-pandemic year, with nearly the entire increase being related to COVID-19 [2]. As a result, review processes were condensed by several journals in order to publish the latest scientific findings in a timely manner [2, 3]. At the same time, a growing number of COVID-related publications has been queried or even retracted due to methodological or ethical concerns [4]. Thus, the quality of reporting of clinical trials is more than ever of utmost importance to determine the quality and relevance of the reported results. Therefore, the *Strengthening the Reporting of Observational Studies in Epidemiology* (STROBE) statement provides a renowned guideline in order to enhance the reporting quality of observational studies [5]. Up to now, no evidence on the quality of reporting of observational studies about treatments for COVID-19 exists. Thus, we aimed to analyse the adherence of the reporting of analytical, observational studies on the treatment of COVID-19 to the STROBE statement. We hypothesised an improvable reporting quality of publications on this topic from the year 2020.

## Methods

### Study design

We performed a retrospective cross-sectional study of scientific publications about observational, analytical clinical studies on the topic of treatment of COVID-19 published during the year of 2020. The entire analysis is reported according to the STROBE checklist [5]. All aspects of systematic literature search and analysis in this study are reported according to the *Preferred Reporting Items for Systematic reviews and Meta-Analyses* (PRISMA) statement [6]. A study protocol was not published.

### Setting

This analysis was initiated and conducted at the Department of Anaesthesiology of the RWTH Aachen University Hospital, from September 2020 to March 2021. We performed a systematic literature search in the United States National Library of Medicine’s Medline database. The query was repeated monthly with the latest search being performed on March 22, 2021.

### Selection of eligible studies

We used PubMed’s search filter on clinical study categories – category: *Therapy*, optimised for *sensitive/broad* search—based on the work of Haymes et al. [7, 8] to search all relevant publications on the treatment of COVID-19 of the entire year 2020 [7, 8]. The resulting search string was adapted to exclude the publication types meta-analysis, review, case report, comment, editorial and letter a priori. The final search term was: ((Therapy/Broad[filter]) AND (Covid-19)) AND (("2020/01/01"[Date—Publication]: "2020/12/31"[Date—Publication])) NOT ("Comment"[Publication Type] OR "Meta-Analysis"[Publication Type] OR "Letter"[Publication Type] OR "Editorial"[Publication Type] OR "Review"[Publication Type] OR "Case Reports"[Publication Type]). Subsequently, one author (IP) screened the titles and abstracts of all results upon eligibility and excluded all non-suitable publication types (interventional studies, reviews, meta-analysis, case reports and case series, opinion articles, guidelines, study protocols and preprints as well as solely descriptive observational studies) and all publications not reporting about the treatment of COVID-19 patients (laboratory or animal studies, studies on risk assessment, prevention). Further, we excluded all studies with less than 100 participants to ascertain the clinical relevance of the included studies. In case of ambiguities regarding the study allocation, decision-making based on the full text of the publication. Uncertainties were discussed with a second author (SZ) and in case of persistent discrepancies a third author (AK) was consulted to make the definite decision. In the final analysis, all publications reporting about analytical, observational studies about the treatment of at least 100 COVID-19 patients were included in the assessment. A sample size calculation was not feasible due to the explorative nature of the study.

### Data extraction

To standardise the interpretation of the STROBE guideline for data extraction, clear requirements for every item of the STROBE checklist were predefined by three authors (AK, IP, SZ) in conformity with the STROBE’s explanation and elaboration document [9]. Based on these definitions, a data sheet was elaborated containing questions for all 22 items. The adherence to the STROBE criteria was assessed by one question for each item without subitem. In case of multiple subitems, each subitem was assessed individually. This resulted in a total of 34 questions that could be answered with *yes* (all requirements fulfilled) or *no* (not all requirements fulfilled). Depending on the individual item, one or more checkpoints were implemented to question all requirements based on the STROBE’s explanation and elaboration document [9]. Each of the 34 questions was only rated as “fulfilled” if all belonging checkpoints were sufficiently reported. The dichotomous rating as well as the necessity to fulfil the requirements of an item in their entity are based on the rationale of Turner et al. [10]. To further distinguish between items not being sufficiently reported and items not mentioned at all a subsequent analysis was added where reasonable. For the following items, the further option *not applicable* was added, since these items do not apply to all studies: 6b – *Matching criteria* (not applicable to study types other than cohort and case–control studies), 12b (not applicable if no subgroup analyses were performed), 12d – *Additional statistical methods* (not applicable in several combinations of study types and/or methodological strategies), 12e – *Sensitivity analyses* (not applicable if no sensitivity analysis was performed), 14c – *Summarised follow-up time* (only applicable to cohort studies with follow-up), 16b – *Category boundaries* (not applicable if no continuous variables were categorised), 17 – *Results of other analyses* (only applicable if additional analyses were performed). The 34-item checklist was then tested by four authors (AK, IP, LG, SZ) with a sample of three cohort, three case–control and three cross-sectional studies in order to reveal possible sources of deviating interpretation among the authors. The data sheet containing all predefined requirements is presented in Additional file 1. Of note, item 16c (Translation of estimates of relative risk into absolute risk) was always rated “not applicable” since the decision, whether it is relevant to translate estimates of relative risk, has to be made by the authors. All STROBE items and sub-items were rated equally. The location of the corresponding author’s institution, the number of participants, the topic as well as the date of publication was taken from each publication. The journals’ impact factors of the year 2019 were extracted from the ISI Web of Knowledge website. The information about the recommendation to use the STROBE statement was retrieved from the author’s guidelines of the respective journal’s websites.

### Bias

To avoid selection bias, we used PubMed’s implemented search strategy for clinical queries with a previously validated sensitivity of 97% [8]. Every uncertainty regarding the correct classification of a publication’s study type was discussed among at least three authors (AK, IP, SZ). After initial analysis of all included publications upon item adherence (IP), each publication was cross-checked by one of two other authors (LG, SZ) independently and inconsistencies were discussed to obtain a consensus. In case of persistent ambiguities, a fourth author (AK) was consulted in order to obtain a final decision. Then, Cohen’s kappa was calculated to assess inter-rater reliability. We did not perform blinding to authors’ and journals’ names, since there is no evidence for a reduced risk of bias when applying this method [10].

### Analysis

The number of sufficiently reported items and its proportion in relation to all applicable items was calculated for each publication. Primary outcome was the percentage of sufficiently reported checklist items of all publications analysed. Secondary outcomes were the average numbers of sufficient reports for each single item and sub-item. Further, the following potential predictors for STROBE guideline adherence were investigated: impact factor, the month of publication, the country of origin, the recommendation to use STROBE and the mention of STROBE adherence in the publication.

### Statistical methods

We calculated the number of and percentage adherence to each individual STROBE item and sub-item for all included observational studies. We computed the median and interquartile range as well as the mean and standard deviation for the summary statistics of the primary endpoint. Normality was assessed by Shapiro–Wilk test and consecutive graphical analysis of quantile–quantile (Q-Q) plots. After testing for homoscedasticity, unpaired, two-sided Student’s t-test or one-way analysis of variance (ANOVA) with Tukey–Kramer post-hoc test was computed to test for differences between two or multiple groups. Multiple linear regression analysis was performed to identify independent predictors for better STROBE checklist adherence. Variables were selected according to prior scientific knowledge. After ensuring all statistical assumptions for linear regression analysis we first performed separate simple linear regression analyses for all prespecified predictor variables. Consecutively, an exploratory multiple linear regression model was fit to model the influence of potential predictors on the percentage adherence to the STROBE checklist. Therefore, the IF was grouped in quartiles and categorical variables were transformed into dummy variables. Publications were excluded from the regression analyses in case of missing values for the IF. A *p-*value of less than 0.05 was considered to be significant. We performed all our statistical analyses using SPSS 27 Statistics Software (IBM Corporation, Armonk, NY, USA).

## Results

### Articles

**Fig. 1:**
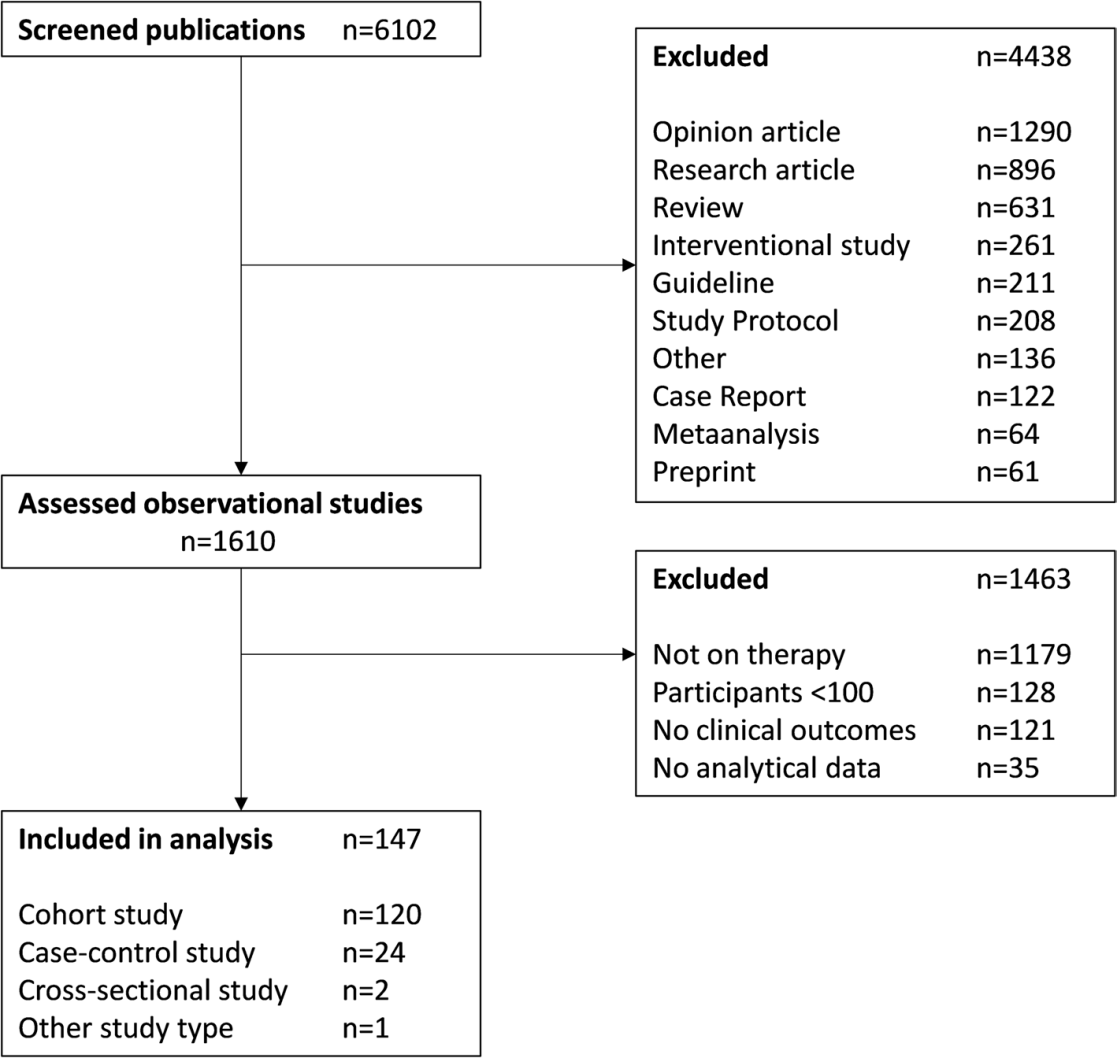
Flowchart**.** Process of screening and inclusion of observational studies for the current study

We screened a total of 6102 articles via our search strategy for the entire year 2020 and identified a total of 1610 observational studies. We consecutively excluded 1463 articles based on our eligibility criteria, see Fig. 1 for details. Among these were publications reporting on less than 100 participants (*n* = 128), reporting no clinical outcomes (*n* = 121) and containing no analytical data (*n* = 35). A detailed list of excluded publications is provided in Additional file 5. The remaining 147 publications reporting analytical data on the therapy of COVID-19 met all eligibility criteria and were included in our analysis. The list of all publications is presented in Additional file 3. They comprised data of 100 to 8075 patients (median 262, IQR 166–764). Articles were published in 96 journals with a median (IQR) impact factor of 3.6390 (2.7395–6.1500). The majority of all publications was written by U.S. American (*n* = 42) and Chinese (*n* = 40), followed Italian (*n* = 21), Spanish (*n* = 14) and French (n = 6) authors. The entire sample comprises a total of 20 nations of origin. Publications reported about the treatment with glucocorticoids (*n* = 25), monoclonal antibodies (*n* = 21), anticoagulants (*n* = 15), antivirals (*n* = 14), antimalarials (*n* = 11), immunomodulators (*n* = 6), combinations of different groups of pharmaceuticals (*n* = 31), other pharmaceuticals (*n* = 16) and ventilation (*n* = 8).

### Main results

**Fig. 2:**
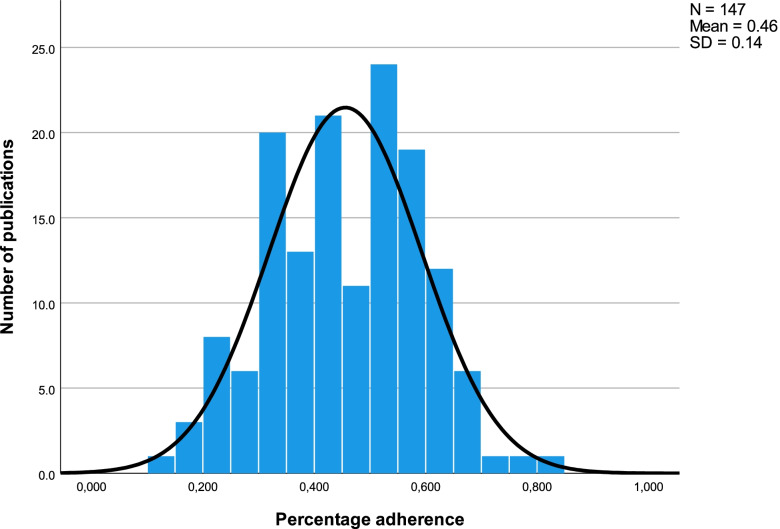
Frequency of percentage adherence**.** Frequency of the percentage adherence to the STROBE checklist items within the sample of 147 publications analysed

The percentage adherence to the STROBE checklist of all 147 included observational studies, reporting analytical data on the treatment of COVID19 patients, is presented in Table 1. Furthermore, we show summary statistics for the adherence of the analysed sample to each individual STROBE item and sub-item in Table 1. The included observational studies reported sufficiently a mean of 45.6% (SD 13.7%) of all analysed STROBE checklist items with a range of 14.2–82.1%, see Fig. 2. The most frequently sufficiently reported items among all publications were items 15 (Outcome data) and 12a (Description of all statistical methods) with rates of 94.6% and 90.5%, respectively. Items 17 (Results of other analyses) and 12e (Description of sensitivity analyses) were reported in 95.2% of 63 applicable publications and 94.4% of 36 publications, respectively. In contrast, items 9 (Methods to address potential sources of bias), 19 (Limitations) and 6a (Eligibility criteria, sources and methods of participant’s selection) were only reported correctly in 1.4%, 3.4% and 6.1% of all publications, respectively. Only 45.6% of all studies sufficiently indicated the study design in a commonly used term, such as “retrospective cohort study”, in its title or abstract (Item 1a) and only 41.5% reported the key elements of the study design in the method’s section as required (Item 4). Most studies sufficiently reported 50–60% of all STROBE items (Fig. 2). A differentiation between insufficiently reported and not being reported at all for each individual item is presented in Additional file 2 where applicable. The list of the percentage adherence of each individual publication is presented in Additional file 3.

### Other analyses

**Fig. 3:**
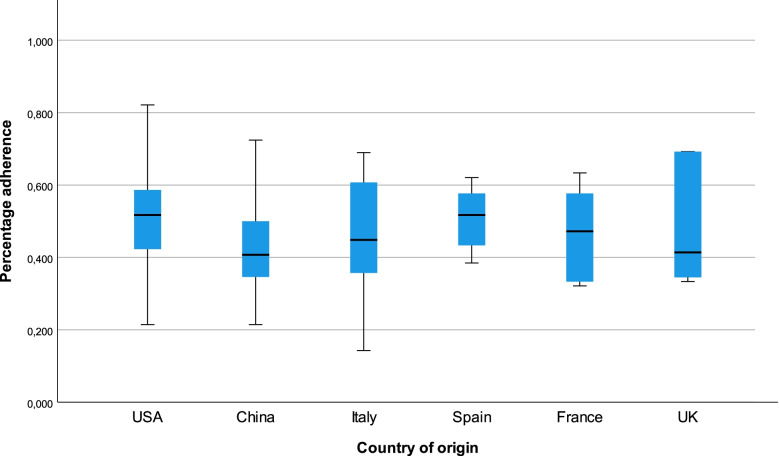
Percentage adherence by country. Mean percentage adherence to the STROBE checklist stratified by country of origin for the top-6 countries. USA: mean 50.2%, *n* = 42; China: mean = 41.1%, *n* = 40; Italy: mean = 45.5%, *n* = 21; Spain: mean = 51.5%, *n* = 14; France: mean = 46.8%, *n* = 6; UK: mean = 49.5%, *n* = 5

U.S. American authors gained a significantly higher percentage adherence to the STROBE checklist (mean 50.2%) compared to Chinese authors (41.1%), mean difference 9.1% (SD 2.8%, 95% CI 0.1 to 17.3%). An overview of the mean percentage adherence by country of origin for the top six countries is presented in Fig. 3. Only 14 publications mentioned the STROBE checklist in their method’s section. These publications showed a significantly higher percentage adherence of 53.5% vs. 44.8% to the STROBE checklist, mean difference 8.7% (SD 3.8%, 95% CI 1.3 to 16.2, *n* = 147). The journals of 57 included publications recommended to use the STROBE checklist in their author’s guidelines. These publications achieved a significantly increased percentage adherence of 51.3% vs. 42.2% to the STROBE checklist, mean difference 9.1% (SD 2.2%, 95% CI 4.8 to 13.4, *n* = 146). Cross-check of item adherence by a second author showed a robust inter-rater reliability of kappa = 0.838. In the multiple linear regression model presented in Table 2 the IF and the recommendation to use STROBE in the author guideline were significant predictors of a higher percentage adherence to the STROBE checklist. The recommendation of STROBE in the author guideline resulted in an increase of 4.76 percentage points (95% CI 0.181 to 9.338) in the percentage adherence. The point estimate for the difference between an IF from the 1^st^ and 4^th^ quartile was 11.07%, 95% CI 5.12 to 17.02). In contrast, publications from Chinese authors were associated with a decrease of 6.65 percentage points (95% CI -12.230 to -1.075) in the percentage adherence compared to US American authors. The separate linear regression models for each individual predictor are presented in Additional file 4.

**Table 1:** Main outcomes

**Main results**	**STROBE Item description **[5]	**Item No**	**n (%) of adhering publications; total *n* = 147**
**Title and abstract**	(*a*) Indicate the study’s design with a commonly used term	1a	67 (45.6)
	(*b*) Provide in the abstract an informative and balanced summary	1b	102 (69.4)
**Introduction**			
Background/ rationale	Explain the scientific background and rationale for the investigation	2	24 (16.3)
Objectives	State specific objectives, including any prespecified hypotheses	3	113 (76.9)
**Methods**			
Study design	Present key elements of study design early in the paper	4	61 (41.5)
Setting	Describe the setting, locations, and relevant dates	5	108 (73.5)
Participants	(*a*) *Cohort study*—Give the eligibility criteria, and the sources and methods of selection of participants and the follow-up *Case–control study*—Give the eligibility criteria, and the sources and methods of case ascertainment and control selection. Give the rationale for the choice of cases and controls *Cross-sectional study*—Give the eligibility criteria, and the sources and methods of selection of participants	6a	9 (6.1)
	(*b*) *Cohort study*—For matched studies, give matching criteria and number of exposed and unexposed *Case–control study*—For matched studies, give matching criteria and the number of controls per case	6b	17/40^a^ (42.5)
Variables	Clearly define all outcomes, exposures, predictors, potential confounders, and effect modifiers. Give diagnostic criteria	7	27 (18.4)
Data sources/ measurement	For each variable of interest, give sources of data and details of methods of assessment (measurement). Describe comparability of assessment methods if there is more than one group	8	117 (79.6)
Bias	Describe any efforts to address potential sources of bias	9	2 (1.4)
Study size	Explain how the study size was arrived at	10	18 (12.2)
Quantitative variables	Explain how quantitative variables were handled in the analyses. Describe which groupings were chosen and why	11	97 (66.0)
Statistical methods	(*a*) Describe all statistical methods, including those used to control for confounding	12a	133 (90.5)
	(*b*) Describe any methods used to examine subgroups and interactions	12b	6/40^a^ (15.0)
	(*c*) Explain how missing data were addressed	12c	41 (27.9)
	(*d*) *Cohort study*—Explain how loss to follow-up was addressed *Case–control study*—Explain how matching of cases and controls was addressed *Cross-sectional study*—Describe analytical methods taking account of sampling strategy	12d	10/30^a^ (33.3)
	(*e*) Describe any sensitivity analyses	12e	34/36^a^ (94.4)
**Results**			
Participants	(*a*) Report numbers of individuals at each stage of study	13a	93 (63.3)
	(*b*) Give reasons for non-participation at each stage	13b	88 (59.9)
	(*c*) Consider use of a flow diagram	13c	61 (41.5)
Descriptive data	(*a*) Give characteristics of study participants and information on exposures and potential confounders	14a	79 (53.7)
	(*b*) Indicate number of participants with missing data for each variable of interest	14b	39 (26.5)
	(*c*) *Cohort study*—Summarise follow-up time	14c	13/26^a^ (50.0)
Outcome data	*Cohort study*—Report numbers of outcome events or summary measures over time *Case–control study—*Report numbers in each exposure category, or summary measures of exposure *Cross-sectional study—*Report numbers of outcome events or summary measures	15	139 (94.6)
Main results	(*a*) Give unadjusted and confounder-adjusted estimates and their precision. Make clear which confounders were adjusted for and why	16a	37 (25.2)
	(*b*) Report category boundaries when continuous variables were categorised	16b	2/3^a^ (66.7)
	(*c*) If relevant, consider translating estimates of relative risk into absolute risk for a meaningful time period	16c	Not evaluated
Other analyses	Report other analyses done	17	60/63^a^ (95.2)
**Discussion**			
Key results	Summarise key results with reference to study objectives	18	35 (23.8)
Limitations	Discuss limitations and potential bias of the study	19	5 (3.4)
Interpretation	Give a cautious overall interpretation of results	20	95 (64.6)
Generalisability	Discuss the generalisability (external validity) of the study results	21	40 (27.2)
**Other Information**			
Funding	Give the source of funding and the role of the funders	22	85 (57.8)
**Overall adherence of all analysed publications**	Mean ± standard deviation	all	45.6 ± 13.7
	Median [interquartile range]	all	46.2 [34.6–57.1]

The present table shows the number and percentage of adherence of the analysed publications to each individual STROBE item as well as the overall percentage adherence to the STROBE checklist. The item names and descriptions are taken from the original STROBE checklist [5]

^a^Indicates number of applicable studies in case that the item was not applicable to all the studies analysed

**Table 2:** Multiple linear regression model for the percentage adherence to the STROBE checklist

**Independent variable**	**Point estimate of change in percentage adherence (Unstandardised coefficient β)**			**Standardised coefficient Beta**	***P*-value**
	**Difference**	**95% CI**	**SE**		
Month of publication	.847	-.177 to 1.872	.518	.130	.104
STROBE mentioned	4.304	-2.808 to 11.415	3.594	.096	.233
STROBE in author guidelines	4.760	.181 to 9.338	2.314	.171	.042
Impact factor ^a^					
2^nd^ quartile	8.633	2.745 to 14.521	2.976	.278	.004
3^rd^ quartile	6.658	.772 to 12.543	2.974	.215	.027
4^th^ quartile	11.072	5.121 to 17.022	3.007	.357	< .001
Country of origin ^b^					
China	-6.653	-12.230 to -1.075	2.819	-.224	.020
Italy	-6.574	-13.383 to .236	3.441	-.168	.058
Spain	1.566	-6.020 to 9.153	3.834	.035	.684
France	-1.927	-12.635 to 8.781	5.411	-.029	.722
Great Britain	.0420	-11.451 to 11.535	5.808	.001	.994
Other countries	-7.838	-14.869 to -.807	3.553	-.195	.029

The present table shows the effects of the prespecified independent variables (predictors) on the percentage adherence to the STROBE checklist according to a multiple linear regression model. Overall regression model: R2 = 0.285; adjusted R2 = 0.217; F (12,127) = 4.214; *p* =  < .001. Dataset *n* = 140; missing values for IF *n* = 7

^a^ Estimates to be interpreted in relation to 1^st^ quartile. Quartile boundaries: 1^st^: 0.717 to 2.739, 2^nd^: 2.740 to 3.639, 3^rd^: 3.656 to 5.893, 4^th^: 6.407 to 74.669

^b^ Estimates to be interpreted in relation to the reference country USA

## Discussion

### Summary

To the best of our knowledge, this study is the first to evaluate the reporting quality of observational studies on the treatment of COVID-19 on full text level. As hypothesised in advance, the reporting quality of the analysed studies was poor throughout the entire year 2020. The 147 analysed studies achieved only a mean percentage adherence of 45.6% to the STROBE checklist. Even though the majority of journals within the present sample recommends reporting in accordance with the STROBE checklist, this led only to a slight increase of the STROBE adherence of 9%.

### Interpretation

Previous works that evaluated the STROBE checklist adherence of publications outside the context of the present pandemic, reported a median adherence to the STROBE items of 59%, 63%, 70% and 83% [11–14]. All of them analysed publications in the top ranked journals of one or more medical disciplines. Even though our data are not directly comparable with these previous analyses limited to a few journals, our results imply remarkable deficits of the analysed literature in terms of reporting quality. This corresponds to the recent findings of Quinn et al., who reported a 12% difference in checklist adherence of COVID-19 publications compared to non-COVID publications in high-Impact journals [15]. The transparent and high-quality reporting of research results is of utmost importance for drawing correct conclusions from the results. It is further indispensable to impede misinterpretation or even for the prevention of fraud. In the context of the COVID pandemic, these issues have gained further importance. As of the end of July 2020, 18 published articles and 14 preprints about COVID-19 have already been retracted or withdrawn [4]. Methodological concerns were the most frequent cause (*n* = 8), followed by deception (*n* = 6) [4]. The discussion about hydroxychloroquine was initially based on the results of observational studies, which were later questioned due to possible biases [15]. Both publications underline the importance of reporting quality. Some of the items of the STROBE checklist are mandatory for high-quality reporting. These include, in our opinion, clear eligibility criteria and sources of participants (item 6a), description of all statistical methods used (item 12a), main results including unadjusted and adjusted estimates (item 16a) and the discussion of limitations including potential bias (item 19). Among these, item 12a was fulfilled in 90.5% of all publications and thus being one of the most frequently fulfilled items in this analysis. This is in line with previous studies, which reported fulfilment rates of 90 to 100% for this item [12, 14]. In contrast, item 19 was only reported in 3.4% of cases indicating a remarkably poor quality of reporting of the limitations, see Additional file 2. Our analysis revealed that this result was specifically caused by a lack of reporting and discussion of potential bias, even though this is considered to be essential by the STROBE authors [9]. A similar result was obtained for the reporting of the eligibility criteria and sources of participants (item 6a). This item was only fulfilled in 6.1% of publications analysed. The main reason was a lack of reporting of the source of the participants, as presented in Additional file 2. Even the central purpose of a publication, the reporting of the results, was affected by a massive lack of reporting quality. Only 25.2% of the publications adhered to the respective item 16a. Our in-depth analysis revealed a multitude of causes, impeding sufficient reporting including imprecise reporting of unadjusted or adjusted estimates, relevant confounders or the exclusion of variables from the analysis, see Additional file 2. The alarming results regarding the above mentioned three items has not been reported before in any other similar analysis of STROBE checklist adherence. Previous data showed rates of adherence of 84–85% for item 6a, 53–60% for item 16a and 55–88% for item 19 respectively [12, 16]. If these findings can be explained by the extraordinary circumstances of the current pandemic, remains unclear. The adherence to the STROBE checklist is not the only, but the most renowned way to ensure high quality reporting of observational studies. Given the fact, that 59.4% of the journals represented in the present analysis recommend the STROBE checklist, it is remarkable, that only 9.5% of the publications reported its application. We extracted the IF of the included publications’ journals and analysed its influence on the percentage adherence to the STROBE checklist on publication level in the linear regression analysis. We acknowledge that the journal’s IF is a measure of the citation frequency of the journal. It should neither be used to estimate the citation frequency of the publication itself nor its quality [17]. Nevertheless, the IF is a common bibliographic measure reflecting the journal’s perception within the scientific community and its reputation. The reporting quality should be a crucial quality characteristic throughout all publications of a journal. Thus, we expected a superior reporting quality of publications in renowned journals. In contrast, our regression analysis shows only a moderate increase of the percentage adherence to STROBE in top ranked journals. Together with the poor overall adherence to the checklist this underlines the need for further efforts to achieve a satisfying reporting quality.

### Methodological considerations

To the best of our knowledge, the present work is the first providing in-depth analysis of the reporting quality according to the STROBE checklist. Previous works, independent of the analysed subset of publications, only questioned the STROBE checklist items themselves. Our elaborated data extraction sheet questioned all checkpoints required by the STROBE’s explanation and elaboration document as requested by the guideline authors who “strongly recommend using the STROBE checklist in conjunction with the explanatory article”. [5, 9]. As an example, Item 9 (Bias) questions the reporting of *Efforts concerning potential sources of bias*, which was reported by 17.7% of all publications analysed, see Additional file 2 for details. In contrast, the STROBE’s explanation and elaboration document additionally demands the *Discussion of likelihood (e.g. direction and magnitude)*. Since only two publications within our analysis addressed this requirement, the overall adherence to item 9 was only 1.4%, see Table 1. Our criteria for fulfilment of the item’s requirements were therefore rather rigorous. In our opinion, this in-depth analysis is a unique strength of our approach of analysis, since it reflects the profound rationale of the STROBE checklist’s authors. The guideline consists of items that the guideline authors declare to be “essential for good reporting of observational studies” and that “should be addressed in sufficient detail” [5]. At the same time, it limits comparability of our results with previous analyses since previous studies could have overestimated the STROBE checklist adherence. We used a dichotomous rating for our analysis differentiating between comprehensive item adherence and non-adherence. The underlying rationale is based on the recommendations of the guideline authors as mentioned above. It is further supported by a Cochrane systematic review evaluating the adherence of publications to the CONSORT statement. The authors explicitly advise to “assess the completeness of reporting of each checklist item in a dichotomous fashion and moreover generally suggest to trial authors that items are only’complete’ when adhered to in their entirety” [10]. Our work was designed as a retrospective observational study collecting new data on the quality of reporting of observational studies. Our search strategy, using PubMed’s implemented search strategy for clinical queries was a systematic, but pragmatic approach to identify the most relevant literature of interest. Its sensitivity has been previously reported to be 97% [8]. Nevertheless, the focus was limited to only one literature database. We did not perform the entire literature search in duplicate, but in case of uncertainties regarding eligibility, up to three authors were involved in the decision-making. This approach to study selection is an adapted version of the “Assessment of records by more than one reviewer” described in the PRISMA 2020 explanation and elaboration publication [18]. The eligibility criteria (study type, analytical data of treatments and number of participants) of our study were clearer to assess compared to those of a systematic review (e.g. thematic consistency, evaluation of the risk of bias). Thus, we believe our methodological approach is sufficient to ascertain a high-quality search. Even though the present study searched and analysed publications, it should not be considered as a systematic review. We did not compile data of multiple publications in order to summarise current evidence in a field of research. Instead, we collected new data on the quality of reporting of observational studies. Due to its nature as an observational study investigating publications based on a systematic literature search, the project was not suitable for registration in neither a clinical trial nor in a review database, i.e. ClinicalTrials.gov or PROSPERO. As we are committed to transparent reporting, we report this work in accordance with the STROBE checklist as far as applicable for our study design. We do not report, for example, the effects of a treatment or the effects of any kind of risk factors. Thus, our study is lacking the typical effect estimates required by STROBE checklist item 16. Since the search strategy, which is figuratively speaking our method of recruitment, should also be transparently reported, we followed the PRISMA statement and addressed several methodological items in addition to the STROBE statement [6]. Nevertheless, key elements of PRISMA regarding extraction of study data, syntheses and their results including effect estimates were not applicable to our study design. Further, we cannot preclude a possible selection bias due to the fact, that some poorly reported studies were unintendedly excluded from the analysis, since their nature as an analytical observational study was not identifiable in the title, abstract or methods section. This would lead to an overestimation of the reporting quality in the sense of the primary endpoint of our study. Finally, we fitted a multiple linear regression model to identify predictors of STROBE item adherence. It must be stated clearly that this additional analysis is only of explorative nature and influenced by inevitable residual confounding.

## Conclusion

In 147 observational, clinical studies on the treatment of COVID-19 published in the first year of the pandemic, we found a poor mean proportion of 45.6% sufficiently reported items of the STROBE checklist. Crucial STROBE items, targeting the correct reporting of the participants, the main outcomes and limitations, were only fulfilled in 6.1%, 25.2% and 3.4% respectively. Further research, applying the same methodology of analysis to other samples of publications apart from the present topic will help to classify our results more precisely.

## Supplementary Information

**Additional file 1. **Data sheet used for analysis and data extraction. The present data sheet is an edited version of the original STROBE checklist [5]. It has been modified in accordance with the STROBE Explanation and Elaboration document [9].

**Additional file 2.** Additional data. Part A shows the number and percentage adherence to each individual checkpoint in case one item or sub-item was questioned by multiple checkpoints to target all requirements based on the STROBE’s explanation and elaboration document [9]. Part B shows differentiated data regarding partial item adherence. Non-adherence is hence divided in the two categories Item partially addressed and Item not addressed.

**Additional file 3.** Overview of publications. The table shows bibliographical data for all included publications as well as the country of origin, the category of treatment and the percentage adherence to the STROBE checklist.

**Additional file 4.** Separate linear regression models for the percentage adherence to the STROBE checklist. The table shows the effects of the prespecified independent variables (predictors) on the percentage adherence to the STROBE checklist according to separate simple and multiple linear regression models for each predictor.

**Additional file 5.** Summary of excluded publications of observational studies on the treatment of COVID-19.

## Data Availability

The template data collection form can be found in Additional file 1. The analytic code is presented in the methods section. The datasets generated during and/or analysed during the current study are available from the corresponding author upon reasonable request.

## Abbreviations

ANOVA: Analysis of variance
CI: Confidence interval
COVID-19: Coronavirus disease 2019
IF: Impact factor
IQR: Interquartile range
RCT: Randomised controlled trial
SARS-CoV-2: Severe acute respiratory syndrome coronavirus2
SD: Standard deviation
STROBE statement: Strengthening the reporting of observational studies in epidemiology statement
Q-Q plot: Quantile–quantile plot
