# Supplementary material for: Poor reporting quality of observational clinical studies comparing treatments of COVID-19 – a retrospective cross-sectional study
Source: BMC Med Res Methodol. 2022 Jan 20;22:23. doi: 10.1186/s12874-021-01501-9 (PMC8771183; doi:10.1186/s12874-021-01501-9)
Supplement: Supplementary file 1 — Additional file 1. Data sheet used for analysis and data extraction. The present data sheet is an edited version of the original STROBE checklist [5]. It has been modified in accordance with the STROBE Explanation and Elaboration document [9]. [file 12874_2021_1501_MOESM1_ESM.docx]

Additional file 1 – Data sheet used for analysis and data extraction

|  | Evaluation criteria | | | Item fulfilled? | | |
| --- | --- | --- | --- | --- | --- | --- |
| Item | Yes | No | Not applicable  (N.a.) | Yes  (1) | No  (0) | N.a. |

Note: Each item can only be rate das “yes” (item fulfilled), if all belonging grey, square checkpoints are marked “yes” or “N.a.”

**Title and abstract**

| 1a | The study’s design is indicated with a frequently used term in title or abstract. | The study design is **not** indicated with a commonly used term in title or abstract. | ___ | ⃝ | ⃝ | - |
| --- | --- | --- | --- | --- | --- | --- |
| 1b  **Abstract** | The abstract provides a balanced summary of what was done and what was found**.**   1. ***Components****: includes research question, description of methods and results, conclusion* 2. ***Information****: includes only information also provided in the main article* 3. ***Numbers****: key results are presented in numerical form*   *(e.g. numbers of participants, estimates of association, measures of uncertainty)* | The abstract does **not** provide a balanced summary of what was done and what was found. | ___ | ⃝ | ⃝ | -  -  -  - |

**Introduction**

| 2  **Background/**  **rationale** | The scientific background and rationale for the investigation are explained in the introduction.   1. *Evidence from* ***previous studies*** *is given with recent pertinent studies* 2. *Evidence from* ***previous studies*** *is given with systematic reviews* 3. ***Gaps of knowledge*** *addressed by this study are given* | The scientific background and rationale for the investigation are **not** explained in the introduction. | ___ | ⃝ | ⃝ | -  -  -  - |
| --- | --- | --- | --- | --- | --- | --- |
| 3  **Objectives** | Specific objectives of the study including pre-specified hypotheses are stated in the introduction. | The objectives or pre-specified hypotheses of the study are **not** stated in the introduction. | ___ | ⃝ | ⃝ | - |

**Methods**

| 4  **Study design** | Key elements of the study design are presented early in the paper.   1. *Presentation of* ***key elements*** *in the methods section or at the end of the introduction* 2. *Explanation of* ***deviations*** *from the three main study types* (case-control study, cohort study, cross-sectional study) | Key elements of the study design are **not** presented early in the paper. | ___   1. *There is no deviation of the study types* | ⃝ | ⃝ | -  - |
| --- | --- | --- | --- | --- | --- | --- |
| 5  **Setting** | Setting, location and relevant dates of the study are described.     1. *Setting (Recruitment sites or sources)* 2. *Location* 3. *Relevant dates (e.g. period of recruitment or data collection, exposure, follow-up)* 4. *Exact dates (not only length of time periods)* | Setting or location of the study are **not** described **or** relevant dates are incomplete. | ___ | ⃝ | ⃝ | -  -  -  -  - |
| 6a  **Participants**  (choose one) | *Cohort study:* Eligibility criteria, sources and methods of selection of participants and methods of follow-up.   1. ***Eligibility criteria****: demographic and clinical* 2. ***Source****: group from which study population was selected* 3. ***Method*** *of* ***recruitment*** 4. ***Methods*** *of* ***follow-up*** | The eligibility **or** sources and methods of the selection of the participants **or** methods of follow-up are **not** described. | Any other study than cohort study.   1. *There is no follow-up due to study design* | ⃝ | ⃝ | ⃝  -  -  - |
|  | *Case-control study:* Eligibility criteria, sources and methods of case ascertainment and control selection and rationale for the choice of cases and controls.   1. ***Eligibility criteria****: demographic and clinical* 2. ***Source****: group from which cases and controls were selected* 3. ***Method*** *used for* ***selection*** *of controls* 4. ***Rationale*** *for choice of cases and controls* | The eligibility criteria are **not** given **or** sources and methods of case ascertainment and control selection are **not** explained **or** the rationale for the choice of cases and controls is **not** given. | Any other study than case-control study. | ⃝ | ⃝ | ⃝  -  -  -  - |
|  | *Cross-sectional study:* Eligibility criteria, sources and methods of selection of participants.   1. ***Eligibility criteria****: demographic and clinical* 2. ***Source****: group from which study population was selected* 3. ***Method*** *used for* ***selection*** *of participants* | The eligibility criteria are **not** given **or** sources and methods of the selection of the participants are **not** explained. | Any other study than cross-sectional study | ⃝ | ⃝ | ⃝  -  -  - |
| 6b  (choose one) | *Cohort study:* For matched studies, matching criteria and number of exposed and unexposed are given.   1. ***Matching criteria*** 2. ***Rationale*** *for choice of matching variables* 3. ***Methods*** *used* *for matching* 4. ***Number*** *of* ***exposed and unexposed*** | Matching criteria **or** number of exposed and unexposed are **not** given. | Any other study than cohort study **or** no matching performed. | ⃝ | ⃝ | ⃝  -  -  -  - |
|  | *Case-control study:* For matched studies, matching criteria and number of controls per case are given.   1. ***Matching criteria*** 2. ***Rationale*** *for choice of matching variables* 3. ***Methods*** *used* *for matching* 4. ***Number*** *of* ***controls per case*** | Matching criteria **or** number of controls per case are **not** given **or** the rationale for the choice of matching variables is not made clear. | Any other study than case-control study **or** no matching performed. | ⃝ | ⃝ | ⃝  -  -  -  - |
| 7  **Variables** | All outcomes, exposures/ predictors, potential confounders and effect modifiers are defined.  Diagnostic criteria are given.   1. ***Variables:*** *definition of* *outcomes, exposures/ predictors, confounders/ effect modifiers* 2. ***Diagnostic criteria:*** *for any disease or event* | The variables are **not** defined **or** diagnostic criteria are **not** given for all diseases or events. | ___ | ⃝ | ⃝ | -  -  - |
| 8  **Data sources/**  **measurement** | For each variable of interest sources of data and details of methods of assessment are given.  Comparability of assessment methods for studies with more than one group is described.   1. ***Sources*** *of data* 2. ***Methods*** *of assessment* 3. ***Comparability*** *of methods for different groups* | Sources of data **or** details of methods of assessment are **not** explained for all variables of interest **or** comparability of assessment methods is **not** described. | ___   1. *Methods do not differ in groups* | ⃝ | ⃝ | -  -  - |
| 9  **Bias** | Efforts concerning potential sources of bias are described.   1. ***Measures*** *taken during the study to reduce the potential of bias* 2. *Discussion* *of* ***likelihood***   *(e.g. direction and magnitude)* | Efforts concerning potential sources of bias are not mentioned **or** one or more of the criteria is missing. | ­­­___ | ⃝ | ⃝ | -  -  - |
| 10  **Study size** | It is explained, how the study size was arrived at.  (e.g. sample size **calculation**, **considerations or circumstances** that determine the study size) | It is **not** described, how the study size was arrived at. | ___ | ⃝ | ⃝ | - |
| 11  **Quantitative Variables** | It is explained, how quantitative variables were handled in the analysis. It is described, which groupings were chosen and why.   1. ***Handling*** *of quantitative variables* 2. *Grouping* ***rationale*** *(why it was applied)* 3. *Grouping* ***method***   *(including number of categories, cut-points)* | The analysis of quantitative variables is **not** explained **or** the grouping of data it is **not** described. | ___   1. *No grouping was performed* 2. *No grouping was performed* | ⃝ | ⃝ | -  - |
| 12a  **Statistical methods** | All statistical methods are explained, including those used to control for confounding.   1. *Explanation of* ***main statistical analyses*** 2. *Explanation of methods to* ***control for confounding*** 3. *Reference and statistical software of* ***non-standard*** *or novel approaches* | Explanation of statistical methods is **not** explained for all methods used **or** one or more of the criteria is missing. | ___   1. *No adjustment performed* 2. *No use of novel approaches* | ⃝ | ⃝ | -  - |
| 12b | All methods used to examine subgroups and interactions are described.   1. *Methods used to examine subgroups* 2. *Methods used to examine interactions* 3. *Explanation if they were planned or arouse during the analysis* | Methods for subgroup examination or interactions are **not** described even though carried out. | No subgroup analyses were performed.   1. *None performed* 2. *None performed* 3. *None performed* | ⃝ | ⃝ | ⃝ |
| 12c | It is explained how missing data were addressed. | It is **not** explained, how missing data were addressed. | ___ | ⃝ | ⃝ | - |
| 12d  (choose one) | *Cohort study:* It is described, how loss to follow-up was addressed in the statistical analyses.  (which **censoring strategies** were used) | It is **not** described how los to follow-up was considered in the statistical analyses. | Any other study than cohort study **or** no follow up performed. | ⃝ | ⃝ | ⃝ |
|  | *Case-control study:* It is explained, how matching of cases and controls was addressed in the statistical analyses. | It is **not** explained, how matching was considered in the statistical analyses. | Any other study than case-control study **or** no matching performed. | ⃝ | ⃝ | ⃝ |
|  | *Cross-sectional study:* Analytical methods considering complex sampling strategy are described. | Analytical methods taking account of complex sampling strategy are **not** described. | No complex sampling strategy was used. | ⃝ | ⃝ | ⃝ |
| 12e | Sensitivity analysis is described. | Sensitivity analysis is **not** described even though it was carried out. | No sensitivity analysis was performed. | ⃝ | ⃝ | ⃝ |

**Results**

| 13a  **Participants** | The numbers of individuals at each stage of the study are reported.   1. *Case-control studies: flow of participants* ***separately*** *for cases and controls* ***and*** *for each type of control groups* | The numbers of individuals at each stage of the study are **not** reported. | ___   1. *Any other study than case-control study* | ⃝ | ⃝ | - |
| --- | --- | --- | --- | --- | --- | --- |
| 13b | Reasons for non-participation are given for each stage. | Reasons for non-participation for each stage are **not** given. | ___ | ⃝ | ⃝ | - |
| 13c | Results are presented in a diagram.  (e.g. flow diagram, if possible) | Results are **not** presented in  a diagram. | ___ | ⃝ | ⃝ | - |
| 14a  **Descriptive data** | Characteristics of study participants and information on exposures and potential confounders are given.   1. ***Characteristics***   *(e.g.* *demographic, clinical, social)*   1. *Information on* ***exposures and confounders*** 2. ***Continuous variables:*** *mean and standard deviation* 3. ***Asymmetrical distributed variables****: median and percentile* 4. ***Variables in few ordered categories:*** *numbers and proportions* | Data of characterization of study participants is missing **or** variables are presented in an insufficient way. | ___   1. *No continuous variables presented* 2. *No asymmetrical variables presented* 3. *No ordered categories presented* | ⃝ | ⃝ | -  -  - |
| 14b | For each variable of interest, the number of individuals with missing data is indicated.   1. ***Missing data*** *for exposures, confounders, important patient characteristics* 2. *Reasons and extent of* ***loss to follow-up*** | The number of individuals with missing data is **not** indicated for each variable of interest **or** loss to follow up is not explained. | ___   1. *No follow up performed* | ⃝ | ⃝ | -  - |
| 14c | *Cohort study:* Follow-up time is summarized.  (at least mean or median time) | The follow-up time is **not** sum.marized **or** does **not** include mean or median. | Any other study than Cohort study **or** no follow up performed. | ⃝ | ⃝ | ⃝ |
| 15  **Outcome data**  (choose one) | *Cohort study:* Numbers of outcome events or summary measures over time are reported. | **No** numbers of outcome events **or** summary measures over time are reported. | Any other study than cohort study. | ⃝ | ⃝ | ⃝ |
|  | *Case-control study:* Numbers in each exposure category or summary measures of exposure are reported. (**Separate** reporting for cases and controls) | **No** numbers of each exposure category **nor** summary measures of exposure are presented **or** cases and controls are **not** separately reported. | Any other study than case-control study. | ⃝ | ⃝ | ⃝ |
|  | *Cross-sectional study:* Numbers of outcome events or summary measures are reported. | **No** numbers of outcome events **or** summary measures are presented. | Any other study than cross-sectional study. | ⃝ | ⃝ | ⃝ |
| 16a  **Main results** | Unadjusted and confounder-adjusted estimates are given.   1. *Presentation of* ***unadjusted estimates*** *together with main data* 2. *Results of* ***adjusted estimates*** *with confidence intervals and number of persons analyzed* 3. *Explanation of* ***potential confounders*** *considered in the analyses* 4. *Rationale for* ***including or excluding variables*** *in the statistical analysis* | Unadjusted **or** confounder-adjusted estimates are **not** given **or** the rationale and precision of the adjusted estimates are **not** given. | ___   1. *No adjustment performed* 2. *No adjustment Performed* 3. *No adjustment performed* | ⃝ | ⃝ | -  - |
| 16b | Category boundaries are reported when continuous variables were categorized. | Category boundaries are **not** reported even though continuous variables were categorized. | No continuous variables were categorized. | ⃝ | ⃝ | ⃝ |
| 16c | If relevant, it was considered translating estimates of relative risk into absolute risk for a meaningful time period. | **___** | No translation of relative risk into absolute risk was performed. | - | - | ⃝ |
| 17  **Other analyses** | Other analyses done are reported.  (e.g. analyses of subgroups, analyses of interactions or sensitivity analyses) | Other analyses done are **not** reported in detail. | No other analyses are performed. | ⃝ | ⃝ | ⃝ |

**Discussion**

| 18  **Key results** | Key results are summarized with reference to the study objectives.   1. *Summary of* ***key results*** 2. *Reference to the* ***study objectives*** | Key results are **not** summarized **or** **not** linked to the study objectives. | ___ | ⃝ | ⃝ | - |
| --- | --- | --- | --- | --- | --- | --- |
| 19  **Limitations** | Limitations of the study are discussed.   1. *Identification of* ***potential bias*** *and discussion of likely direction and magnitude* 2. *Discussion of* ***imprecision or further limitations***   *(e.g. study size or measurements*) | Limitations or bias of the study are **not** mentioned. | ___ | ⃝ | ⃝ | -  -  - |
| **20**  **Interpretation** | An overall interpretation is given.   1. ***Interpretation***   *(e.g. consideration of nature of the study, residual confounding, multiplicity of analyses, sensitivity analyses, overall impression)*   1. *Discussion of existing* ***external evidence*** 2. *Explanation of how the* ***new study affects the existing body of evidence*** | An overall interpretation is **not** given **or** one or more of the criteria is missing. | ___ | ⃝ | ⃝ | -  -  -  - |
| 21  **Generalisa-bility** | The generalisability (external validity) of the findings is discussed. | The generalisability of the findings is **not** mentioned. | ___ | ⃝ | ⃝ | - |

**Other information**

| 22  **Funding** | The source of funding and the role of the funders for the present study and original study on which the article is based are given.   1. ***Source*** *of funding and other influence* 2. ***Role*** *of funders* | The source of funding is **not** indicated **or** the role of the funders for the investigation are not clearly defined **or** other sources of influence are **not** described although evidently existing. | ___   1. *There are no sources of funding* | ⃝ | ⃝ | -  - |
| --- | --- | --- | --- | --- | --- | --- |
