# Supplementary material for: Poor reporting quality of observational clinical studies comparing treatments of COVID-19 – a retrospective cross-sectional study
Source: BMC Med Res Methodol. 2022 Jan 20;22:23. doi: 10.1186/s12874-021-01501-9 (PMC8771183; doi:10.1186/s12874-021-01501-9)
Supplement: Supplementary file 2 — Additional file 2. Additional data. Part A shows the number and percentage adherence to each individual checkpoint in case one item or sub-item was questioned by multiple checkpoints to target all requirements based on the STROBE’s explanation and elaboration document [9]. Part B shows differentiated data regarding partial item adherence. Non-adherence is hence divided in the two categories Item partially addressed and Item not addressed. [file 12874_2021_1501_MOESM2_ESM.docx]

Part A – Additional data for items with multiple checkpoints

|  | Item description | n (%) of adhering publications; total n = 147 | | n (%) of non-adhering publications | | n (%) of n.a. |
| --- | --- | --- | --- | --- | --- | --- |
| Title and abstract | **Item 1(b):** Provide in the abstract an informative and balanced summary  *1. Components: includes research question, description of methods and results, conclusion*  *2. Information: includes only information provided in the main article*  *3. Numbers: key results are presented in numerical form* | 102 (69.4)  *119 (81.0)*  *138 (93.9)*  *127 (86.4)* | | 45 (30.6)  *28 (19.0)*  *9 (6.1)*  *20 (13.6)* | | -  *-*  *-*  *-* |
| **Introduction** |  |  | |  | |  |
| Background/ rationale | **Item 2:** Explain the scientific background and rationale for the investigation  *1. Knowledge of previous studies is given with recent pertinent studies*  *2. Knowledge of previous studies is given with systematic reviews*  *3. Gaps of knowledge addressed by this study are given* | 24 (16.3)  *129 (87.8)*  *30 (20.4)*  *103 (70.1)* | | 123 (83.7)  *18 (12.2)*  *117 (79.6)*  *44 (29.9)* | | -  *-*  *-*  *-* |
| **Methods** |  |  | |  | |  |
| Study design | **Item 4:** Present key elements of study design early in the paper  *1. Presentation of key elements in the methods section or at the end of the introduction*  *2. Explanation of deviations of the three main study types* | 61 (41.5)  *62 (42.2)*  *2/3* (66.7)* | | 86 (58.5)  *85 (57.8)*  *1/3* (33.3)* | | -  *-*  144 |
| Setting | **Item 5:** Describe the setting, locations, and relevant dates  *1. Setting (Recruitment sites or sources)*  *2. Location*  *3. Relevant dates (e.g. period of recruitment or data collection, exposure, follow-up)*  *4. Date format (stated with dates, not length of time periods)* | 108 (73.5)  *125 (85.0)*  *128 (87.1)*  *135 (91.8)*  *144 (98.0)* | | 39 (26.5)  *22 (15.0)*  *19 (12.9)*  *12 (8.2)*  *3 (2.0)* | | -  *-*  *-*  *-*  *-* |
| Participants | **Item 6(a):** *Cohort study*—Give the eligibility criteria, and the sources and methods of selection of participants and follow-up  *Case-control study*—Give the eligibility criteria, and the sources and methods of case ascertainment and control selection. Give the rationale for the choice of cases and controls  *Cross-sectional study*—Give the eligibility criteria, and the sources and methods of selection of participants  *1. Eligibility criteria (demographic and clinical)*  *2. Source: group from which study population* ***OR*** *cases and controls were selected*  *3. Methods of recruitment* ***OR*** *selection of controls*  *4. (N.a. for cross-sectional studies) Method of follow up* ***OR*** *rationale for choice of cases and controls* | 9 (6.1)  *121 (82.3)*  *12 (8.2)*  *77 (52.4)*  *21/46* (45.7)* | | 138 (93.9)  *26 (17.7)*  *135 (91.8)*  *70 (47.6)*  *25/46* (54.3)* | | -  *-*  *-*  *-*  *101* |
|  | **Item 6(b):** *Cohort study*—For matched studies, give matching criteria and number of exposed and unexposed  *Case-control study*—For matched studies, give matching criteria and the number of controls per case  (N.a. for cross-sectional studies)  *1. Matching criteria*  *2. Rationale for choice of matching variables*  *3. Methods used for matching*  *4. Number of exposed and unexposed* ***OR*** *controls per case* | 17/40* (42.5)  *36/40* (90.0)*  *20/40* (50.0)*  *33/40* (82.5)*  *36/40* (90.0)* | | 23/40* (57.5)  *4/40* (10.0)*  *20/40* (50.0)*  *7/40* (17.5)*  *4/40* (10.0)* | | 107  *-*  *-*  *-*  *-* |
| Variables | **Item 7:** Clearly define all outcomes, exposures, predictors, potential confounders, and effect modifiers. Give diagnostic criteria  *1. Variables: definition of outcomes, exposures/ predictors, confounders/ effect modifiers*  *2. Diagnostic criteria: for any disease or event* | 27 (18.4)  *28 (19.0)*  *123 (83.7)* | | 120 (81.6)  *119 (81.0)*  *24 (16.3)* | | -  *-*  *-* |
| Data sources/ measurement | **Item 8:** For each variable of interest, give sources of data and details of methods of assessment (measurement). Describe comparability of assessment methods if there is more than one group  *1. Sources of data*  *2. Methods of assessment*  *3. Comparability* | 117 (79.6)  *121 (82.3)*  *125 (85.0)*  *4/4* (100,0)* | | 30 (20.4)  *26 (17.7)*  *22 (15.0)*  *0* | | -  *-*  *-*  *143* |
| Bias | **Item 9:** Describe any efforts to address potential sources of bias  *1. Measures taken during the study to reduce the potential of bias*  *2. Discussion of likelihood (direction and magnitude)* | 2 (1.4)  *26 (17.7)*  *2 (1.4)* | | 145 (98.6)  *121 (82.3)*  *145 (98.6)* | | -  *-*  *-* |
| Quantitative variables | **Item 11:** Explain how quantitative variables were handled in the analyses. Describe which groupings were chosen and why  *1. Handling of quantitative variables*  *2. Grouping rationale (why it was applied)*  *3. Grouping method (including number of categories, cut-points)* | 97 (66.0)  *105 (71.4)*  *10/25* (40.0)*  *20/25* (80.0)* | | 50 (34.0)  *42 (28.6)*  *15/25* (60.0)*  *5/25* (20.0)* | | -  *-*  *122*  *122* |
| Statistical methods | **Item 12(a):** Describe all statistical methods, including those used to control for confounding  *1. Explanation of main statistical analyses*  *2. Explanation of methods to control for confounding*  *3. Reference and statistical software for non-standard or novel approaches* | 133 (90.5)  *141 (95.9)*  *98/107* (91.6)*  *3/3* (100.0)* | | 14 (9.5)  *6 (4.1)*  *9/107* (8.4)*  *0* | | -  *-*  *40*  *144* |
|  | **Item 12(b):** Describe any methods used to examine subgroups and interactions  *1. Methods used to examine subgroups*  *2. Methods used to examine interactions*  *3. Explanation if they were planned or arouse during the analysis* | 6/40* (15.0)  *28/38* (73.7)*  *3/3* (100.0)*  *6/40* (15.0)* | | 34/40* (85.0)  *10/38* (26.3)*  *0*  *34/40* (85.0)* | | 107  *109*  *144*  *107* |
| **Results** |  |  | |  | |  |
| Participants | **Item 13(a):** Report numbers of individuals at each stage of study  *1. Case-control studies: flow of participants separately for cases and controls and for each type of control group* | 93 (63.3)  *5/11* (45.0)* | | 54 (36.7)  *6/11* (54.0)* | | -  *136* |
| Descriptive data | **Item 14(a):** Give characteristics of study participants and information on exposures and potential confounders  *1. Characteristics (e.g. demographic, clinical, social)*  *2. Information on exposures and confounders*  *3. Continuous variables: mean and standard deviation*  *4. Asymmetrical distributed variables: median and percentile*  *5. Variables in few ordered categories: numbers and percentages* | 79 (53.7)  *147 (100.0)*  *132 (89.8)*  *74/134* (55.2)*  *104/109* (95.4)*  *137/141* (97.2)* | | 68 (46.3)  *0*  *15 (10.2)*  *60/134* (44.8)*  *5/109* (4.6)*  *4/141* (2.8)* | | -  *-*  *-*  *13*  *38*  *6* |
|  | **Item 14(b):** Indicate number of participants with missing data for each variable of interest  *1. Missing data for exposures, confounders, important patient characteristics*  *2. Reasons and extent of loss to follow-up* | 39 (26.5)  *44 (29.9)*  *8/37* (21.6)* | | 108 (73.5)  *103 (70.1)*  *29/37* (78.4)* | | -  *-*  *110* |
| Main results | **Item 16(a):** Give unadjusted and confounder-adjusted estimates and their precision. Make clear which confounders were adjusted for and why  *1. Presentation of unadjusted estimates together with main data*  *2. Results of adjusted estimates with confidence intervals and number of persons analysed*  *3. Explanation of potential confounders considered in the analyses*  *4. Rationale for including or excluding variables in the statistical analysis* | 37 (25.2)  *81 (55.1)*  *56/107* (52.3)*  *82/107* (76.6)*  *31/107* (29.0)* | | 110 (74.8)  *66 (44.9)*  *51/107* (47.7)*  *25/107* (23.4)*  *76/107* (71.0)* | | -  *-*  *40*  *40*  *40* |
| **Discussion** |  |  | |  | |  |
| Key results | **Item 18:** Summarise key results with reference to study objectives  *1. Summary of key results*  *2. Reference to the study objectives* | 35 (23.8)  *143 (97.3)*  *35 (23.8)* | | 112 (76.2)  *4 (2.7)*  *112 (76.2)* | | -  *-*  *-* |
| Limitations | **Item 19:** Discuss limitations and potential bias of the study  *1. Identification of potential bias and discussion of likely direction and magnitude*  *2. Discussion of imprecision or further limitations (e.g. study size or measurements)* | 5 (3.4)  *5 (3.4)*  *136 (92.5)* | | 142 (96.6)  *142 (96.6)*  *11 (7.5)* | | -  *-*  *-* |
| Interpretation | **Item 20:** Give a cautious overall interpretation of results  *1. Interpretation (e.g. consideration of the nature of the study, residual confounding, multiplicity of analyses, sensitivity analyses, overall impression)*  *2. Discussion of existing external evidence*  *3. Explanation of how the new study affects existing body of evidence* | 95 (64.6)  *105 (71.4)*  *143 (97.3)*  *127 (86.4)* | | 52 (35.4)  *42 (28.6)*  *4 (2.7)*  *20 (13.6)* | | -  *-*  *-*  *-* |
| **Other Information** | |  |  | |  | |
| Funding | **Item 22:** Give the source of funding and the role of the funders  *1. Source of funding and other influence*  *2. Role of funders* | 85 (57.8)  *125 (85.0)*  *20/61* (32.8)* | | 62 (42.2)  *22 (15.0)*  *41/61* (67.2)* | | -  *-*  *86* |
| * indicates number of applicable studies in case that the item was not applicable to all the studies analysed.  Items and descriptions are extracted from and based on the STROBE Explanation and Elaboration document [9]. | | | | | | |

Part B – Differentiated data regarding partial item adherence

|  | Item description | n (%) of  adhering  publications  -  total n = 147 | | n (%) of  non-adhering  publications  -  item partially addressed | | n (%) of non-adhering  publications  -  item not addressed | | N.a. |
| --- | --- | --- | --- | --- | --- | --- | --- | --- |
| Title and abstract | **Item 1(a):** Indicate the study’s design with a commonly used term in the title or the abstract | 67 (45.6) | | 49 (33.3) | | 31 (21.1) | | - |
|  | **Item 1(b):** Provide in the abstract an informative and balanced summary of what was done and what was found | 102 (69.4) | | 44 (29.9) | | 1 (0.7) | | - |
| **Introduction** |  |  | |  | |  | |  |
| Background/ rationale | **Item 2:** Explain the scientific background and rationale for the investigation being reported | 24 (16.3) | | 117 (79.6) | | 6 (4.1) | | - |
| Objectives | **Item 3:** State specific objectives, including any prespecified hypotheses | 113 (70.1) | | 15 (10.2) | | 19 (12.9) | | - |
| **Methods** |  |  | |  | |  | |  |
| Study design | **Item 4:** Present key elements of study design early in the paper | 61 (41.5) | | 1 (0.7) | | 85 (57.8) | | - |
| Setting | **Item 5:** Describe the setting, locations, and relevant dates, including periods of recruitment, exposure, follow-up, and data collection | 108 (73.5) | | 38 (25.9) | | 1 (0.7) | | - |
| Participants | **Item 6(a):**  *Cohort study*—Give the eligibility criteria, and the sources and methods of selection of participants. Describe methods of follow-up  *Case-control study*—Give the eligibility criteria, and the sources and methods of case ascertainment and control selection. Give the rationale for the choice of cases and controls  *Cross-sectional study*—Give the eligibility criteria, and the sources and methods of selection of participants | 9 (6.1) | | 123 (83.7) | | 15 (10.2) | | - |
|  |  |  | |  | |  | |  |
|  | **Item 6(b):**  *Cohort study*—For matched studies, give matching criteria and number of exposed and unexposed  *Case-control study*—For matched studies, give matching criteria and the number of controls per case  *Case-control study*—Not applicable | 17/40* (42.5) | | 22/40* (55.0) | | 1/40* (2.5) | | 107 |
| Variables | **Item 7:** Clearly define all outcomes, exposures, predictors, potential confounders, and effect modifiers. Give diagnostic criteria, if applicable | 27 (18.4) | | 99 (67.3) | | 21 (14.3) | | - |
| Data sources/ measurement | **Item 8:** For each variable of interest, give sources of data and details of methods of assessment (measurement). Describe comparability of assessment methods if there is more than one group | 117 (79.6) | | 12 (8.2) | | 18 (12.2) | | - |
| Bias | **Item 9:** Describe any efforts to address potential sources of bias | 2 (1.4) | | 24 (16.3) | | 121 (82.3) | | - |
| Study size | **Item 10:** Explain how the study size was arrived at | 18 (12.2) | | 3 (2.0) | | 126 (85.7) | |  |
| Quantitative variables | **Item 11:** Explain how quantitative variables were handled in the analyses. If applicable, describe which groupings were chosen and why | 97 (66.0) | | 14 (9.5) | | 36 (24.5) | | - |
| Statistical methods | **Item 12(a):** Describe all statistical methods, including those used to control for confounding | 133 (90.5) | | 11 (7.5) | | 3 (2.0) | | - |
|  | **Item 12(b):** Describe any methods used to examine subgroups and interactions | 6/40* (15.0) | | 24/40* (60.0) | | 10/40* (25.0) | | 107 |
|  | **Item 12(c):** Explain how missing data were addressed | 41 (27.9) | | not applicable | | 106 (72.1) | | - |
|  | **Item 12(d):**  *Cohort study*—If applicable, explain how loss to follow-up was addressed  *Case-control study*—If applicable, explain how matching of cases and controls was addressed  *Cross-sectional study*—If applicable, describe analytical methods taking account of sampling strategy | 10/30* (33.3) | | not applicable | | 20/30* (66.6) | | 117 |
|  | **Item 12(e):** Describe any sensitivity analyses | 34/36* (94.4) | | not applicable | | 2/36* (5.6) | | 111 |
| **Results** |  |  | |  | |  | |  |
| Participants | **Item 13(a):** Report numbers of individuals at each stage of study | 93 (63.3) | | 1 (0.7) | | 53 (36.1) | | - |
|  | **Item 13(b):** Give reasons for non-participation at each stage | 88 (59.9) | | 4 (2.7) | | 55 (37.4) | | - |
|  | **Item 13(c):** Consider use of a flow diagram | 61 (41.5) | | not applicable | | 86 (58.5) | | - |
| Descriptive data | **Item 14(a):** Give characteristics of study participants and information on exposures and potential confounders | 79 (53.7) | | 68 (46.3) | | 0 (0.0) | | - |
|  | **Item 14(b):** Indicate the number of participants with missing data for each variable of interest | 39 (26.5) | | 6 (4.1) | | 102 (69.4) | | - |
|  | **Item 14(c):** *Cohort study*—Summarise follow-up time | 13/26* (50.0) | | not applicable | | 13/26* (50.0) | | 121 |
| Outcome data | **Item 15:**  *Cohort study*—Report numbers of outcome events or summary measures over time  *Case-control study—*Report numbers in each exposure category, or summary measures of exposure  *Cross-sectional study—*Report numbers of outcome events or summary measures | 139 (94.6) | | 8 (5.4) | | 0 (0.0) | | - |
| Main results | **Item 16(a):** Give unadjusted estimates and, if applicable, confounder-adjusted estimates and their precision. Make clear which confounders were adjusted for and why they were included | 37 (25.2) | | 87 (59.2) | | 23 (15.6) | | - |
|  | **Item 16(b):** Report category boundaries when continuous variables were categorized | 2/3* (66.6) | | 0/3* (0.0) | | 1/3* (33.3) | | 144 |
|  | **Item 16(c):** If relevant, consider translating estimates of relative risk into absolute risk for a meaningful time period | not evaluated | | not evaluated | | not evaluated | | - |
| Other analyses | **Item 17:** Report other analyses done | 60/63* (95.2) | | not applicable | | 3/63* (4.8) | | 84 |
| **Discussion** |  |  | |  | |  | |  |
| Key results | **Item 18:** Summarise key results with reference to study objectives | 35 (23.8) | | 108 (73.5) | | 4 (2.7) | | - |
| Limitations | **Item 19:** Discuss limitations of the study, taking into account sources of potential bias or imprecision. Discuss both direction and magnitude of any potential bias | 5 (3.4) | | 131 (89.1) | | 11 (7.5) | | - |
| Interpretation | **Item 20:** Give a cautious overall interpretation of results considering objectives, limitations, multiplicity of analyses, results from similar studies, and other relevant evidence | 95 (64.6) | | 52 (35.4) | | 0 (0.0) | | - |
| Generalisability | **Item 21:** Discuss the generalisability (external validity) of the study results | 40 (27.2) | | 7 (4.8) | | 100 (68.0) | | - |
| **Other Information** | |  |  | |  | |  | |
| Funding | **Item 22:** Give the source of funding and the role of the funders for the present study and, if applicable, for the original study on which the present article is based | 85 (57.8) | | 40 (27.2) | | 22 (15.0) | | - |
| * indicates number of applicable studies in case that the item was not applicable to all the studies analysed  Items and descriptions are extracted from and based on the STROBE Explanation and Elaboration document {Vandenbroucke, 2007 #97}. | | | | | | | | |
