# Supplementary material for: Poor reporting quality of observational clinical studies comparing treatments of COVID-19 – a retrospective cross-sectional study
Source: BMC Med Res Methodol. 2022 Jan 20;22:23. doi: 10.1186/s12874-021-01501-9 (PMC8771183; doi:10.1186/s12874-021-01501-9)
Supplement: Supplementary file 3 — Additional file 3. Overview of publications. The table shows bibliographical data for all included publications as well as the country of origin, the category of treatment and the percentage adherence to the STROBE checklist. [file 12874_2021_1501_MOESM3_ESM.pdf]

Additional file 3 – Overview of publications

PMID	Author	Journal	DOI	Country of origin	Number of participants	Topic	Percentage adherence
32220112	N. Tang, et al.	J Thromb Haemost	10.1111/jth.14817	China	449	Anticoagulation	35.71%
32297985	X. Shi, et al.	J Med Virol	10.1002/jmv.25893	China	184	Antivirals	30.77%
32379955	J. Geleris, et al.	N Engl J Med	10.1056/NEJMoa2012410	USA	1446	Antimalarials	51.61%
32392282	E. S. Rosenberg, et al.	Jama	10.1001/jama.2020.8630	USA	1438	Combination of pharmaceuticals	75.86%
32409486	M. Mahévas, et al.	Bmj	10.1136/bmj.m1844	France	181	Antimalarials	63.33%
32418114	B. Yu, et al.	Sci China Life Sci	10.1007/s11427-020-1732-2	China	550	Antimalarials	23.08%
32427279	R. Fadel, et al.	Clin Infect Dis	10.1093/cid/ciaa601	USA	213	Glucocorticoids	53.33%
32430428	D. Yan, et al.	Eur Respir J	10.1183/13993003.00799-2020	China	120	Antivirals	42.31%
32445881	P. Xu, et al.	Microbes Infect	10.1016/j.micinf.2020.05.012	China	141	Combination of pharmaceuticals	50.00%
32476080	L. Ayerbe, et al.	J Thromb Thrombolysis	10.1007/s11239-020-02162-z	UK	2075	Anticoagulation	34.48%
32492205	T. Yu, et al.	J Med Virol	10.1002/jmv.26129	China	129	Antivirals	34.62%
32496422	M. Yuan, et al.	Shock	10.1097/shk.0000000000001574	China	132	Glucocorticoids	33.33%
32553536	C. C. Price, et al.	Chest	10.1016/j.chest.2020.06.006	USA	239	Monoclonal antibodies	62.07%
32554861	Y. Huang, et al.	Aging (Albany NY)	10.18632/aging.103370	China	238	Combination of pharmaceuticals	21.43%
32565309	X. Li, et al.	Pharmacol Res	10.1016/j.phrs.2020.105036	China	151	Antivirals	38.46%
32569363	G. Rojas-Marte, et al.	Qjm	10.1093/qjmed/hcaa206	USA	193	Monoclonal antibodies	30.77%
32570043	L. Quartuccio, et al.	J Clin Virol	10.1016/j.jcv.2020.104444	Italy	111	Monoclonal antibodies	39.29%
32571831	A. Fernández-Cruz, et al.	Antimicrob Agents Chemother	10.1128/aac.01168-20	Spain	462	Glucocorticoids	62.07%
32593867	J. C. Lagier, et al.	Travel Med Infect Dis	10.1016/j.tmaid.2020.101791	France	3737	Combination of pharmaceuticals	33.33%
32621621	C. Yu, et al.	J Med Virol	10.1002/jmv.26260	China	128	Antivirals	41.38%
32623082	S. Arshad, et al.	Int J Infect Dis	10.1016/j.ijid.2020.06.099	USA	2541	Combination of pharmaceuticals	51.85%
32634602	F. Bani-Sadr, et al.	Int J Antimicrob Agents	10.1016/j.ijantimicag.2020.106077	France	257	Glucocorticoids	32.14%
32652164	R. Rossotti, et al.	J Infect	10.1016/j.jinf.2020.07.008	Italy	222	Monoclonal antibodies	14.29%
32687645	M. Kelly, et al.	Br J Clin Pharmacol	10.1111/bcp.14482	Ireland	134	Combination of pharmaceuticals	15.38%
32699031	D. R. Rivera, et al.	Cancer Discov	10.1158/2159-8290.Cd-20-0941	USA	2186	Combination of pharmaceuticals	50.00%
32706859	S. A. Olender, et al.	Clin Infect Dis	10.1093/cid/ciaa1041	USA	1130	Antivirals	53.57%
32707096	N. Wang, et al.	Cell Host Microbe	10.1016/j.chom.2020.07.005	China	446	Immunomodulators	57.69%
32712334	S. Tong, et al.	Int J Antimicrob Agents	10.1016/j.ijantimicag.2020.106114	China	115	Antivirals	53.85%
32713677	L. M. Canziani, et al.	J Autoimmun	10.1016/j.jaut.2020.102511	Italy	128	Monoclonal antibodies	35.71%
32720702	T. Maeda, et al.	J Med Virol	10.1002/jmv.26365	USA	224	Monoclonal antibodies	46.15%
32721528	P. Sinha, et al.	Int J Infect Dis	10.1016/j.ijid.2020.07.023	USA	255	Monoclonal antibodies	42.86%
32728438	S. W. X. Ong, et al.	Clin Transl Immunology	10.1002/cti2.1159	Singapore	168	Immunomodulators	50.00%
32732245	M. Scarsi, et al.	Ann Rheum Dis	10.1136/annrheumdis-2020-217712	Italy	262	Other pharmaceuticals	39.29%
32736237	Y. Hu, et al.	Biomed Pharmacother	10.1016/j.biopha.2020.110529	China	308	Glucocorticoids	34.62%
32740371	G. Gao, et al.	J Acquir Immune Defic Syndr	10.1097/qai.0000000000002452	China	129	Combination of pharmaceuticals	23.08%
32748578	S. R. Hao, et al.	J Zhejiang Univ Sci B	10.1631/jzus.B2000211	China	104	Combination of pharmaceuticals	42.86%
32755653	B. Davido, et al.	Int J Antimicrob Agents	10.1016/j.ijantimicag.2020.106129	France	132	Combination of pharmaceuticals	44.44%
32763357	M. Kalligeros, et al.	J Glob Antimicrob Resist	10.1016/j.jgar.2020.07.018	USA	108	Antimalarials	21.43%
32767353	A. De Giorgi, et al.	Eur Rev Med Pharmacol Sci	10.26355/eurrev_202008_22511	Italy	151	Antivirals	34.62%

32772069	B. C. Nelson, et al.	Clin Infect Dis	10.1093/cid/ciaa1163	USA	117	Glucocorticoids	53.57%
32776534	A. Giacomelli, et al.	J Med Virol	10.1002/jmv.26407	Italy	172	Combination of pharmaceuticals	46.15%
32784192	S. Roomi, et al.	J Med Internet Res	10.2196/21758	USA	176	Combination of pharmaceuticals	42.31%
32790733	A. Ip, et al.	PLoS One	10.1371/journal.pone.0237693	USA	547	Combination of pharmaceuticals	57.69%
32791263	J. Tian, et al.	Pharmacol Res	10.1016/j.phrs.2020.105127	China	721	Traditional Chinese medicine	51.85%
32795143	Y. Li, et al.	Emerg Microbes Infect	10.1080/22221751.2020.1807885	China	119	Glucocorticoids	42.31%
32795897	M. Wu, et al.	Int Immunopharmacol	10.1016/j.intimp.2020.106873	China	334	Immunomodulators	39.29%
32798660	T. Klopfenstein, et al.	Int J Infect Dis	10.1016/j.ijid.2020.08.024	France	206	Monoclonal antibodies	57.69%
32804611	M. J. Keller, et al.	J Hosp Med	10.12788/jhm.3497	USA	1806	Glucocorticoids	58.62%
32804790	A. C. Hernandez-Romieu, et al.	Crit Care Med	10.1097/ccm.0000000000004600	USA	231	Ventilation	60.71%
32817707	M. Mikulska, et al.	PLoS One	10.1371/journal.pone.0237831	Italy	196	Combination of pharmaceuticals	44.83%
32835257	G. Guaraldi, et al.	Lancet Rheumatol	10.1016/s2665-9913(20)30173-9	Italy	544	Monoclonal antibodies	64.29%
32838323	N. Biran, et al.	Lancet Rheumatol	10.1016/s2665-9913(20)30277-0	USA	764	Monoclonal antibodies	63.33%
32847947	M. Patel, et al.	BMJ Open Respir Res	10.1136/bmjresp-2020-000650	USA	104	Ventilation	40.74%
32852338	J. F. Mather, et al.	Am J Gastroenterol	10.14309/ajg.0000000000000832	USA	878	Other pharmaceuticals	37.93%
32853673	L. Catteau, et al.	Int J Antimicrob Agents	10.1016/j.ijantimicag.2020.106144	Belgium	8075	Antimalarials	41.38%
32857301	H. Rahmani, et al.	Daru	10.1007/s40199-020-00369-2	Iran	213	Combination of pharmaceuticals	28.57%
32859477	A. D. Castelnuovo, et al.	Eur J Intern Med	10.1016/j.ejim.2020.08.019	Italy	3451	Antimalarials	62.07%
32860964	J. Rodríguez-Baño, et al.	Clin Microbiol Infect	10.1016/j.cmi.2020.08.010	Spain	778	Combination of pharmaceuticals	51.72%
32872629	F. Albani, et al.	J Clin Med	10.3390/jcm9092800	Italy	1376	Combination of pharmaceuticals	53.57%
32880390	J. Wu, et al.	J Clin Endocrinol Metab	10.1210/clinem/dgaa627	China	1763	Glucocorticoids	48.15%
32881982	A. Rodríguez-Molinero, et al.	PLoS One	10.1371/journal.pone.0238681	Spain	418	Other pharmaceuticals	51.72%
32882767	E. J. Kim, et al.	Yonsei Med J	10.3349/ymj.2020.61.9.826	South Korea	110	Antivirals	25.00%
32885463	J. Ferguson, et al.	J Clin Pharmacol	10.1002/jcph.1749	USA	141	Anticoagulation	27.59%
32903258	M. Majmundar, et al.	PLoS One	10.1371/journal.pone.0238827	USA	205	Glucocorticoids	57.69%
32911000	M. Schiavone, et al.	Int J Cardiol	10.1016/j.ijcard.2020.09.001	Italy	844	Anticoagulation	42.86%
32926319	Z. Shu, et al.	Front Med	10.1007/s11684-020-0803-8	China	293	Traditional Chinese medicine	34.62%
32926320	G. Chen, et al.	Front Med	10.1007/s11684-020-0813-6	China	312	Traditional Chinese medicine	59.26%
32926573	M. Lauriola, et al.	Clin Transl Sci	10.1111/cts.12860	Italy	377	Combination of pharmaceuticals	50.00%
32930657	P. M. Carlucci, et al.	J Med Microbiol	10.1099/jmm.0.001250	USA	932	Antivirals	34.62%
32941741	J. Chen, et al.	Expert Rev Respir Med	10.1080/17476348.2020.1822741	China	200	Combination of pharmaceuticals	40.74%
32941927	R. Huang, et al.	Eur J Pharmacol	10.1016/j.ejphar.2020.173556	China	309	Glucocorticoids	35.71%
32946668	M. R. Pereira, et al.	Am J Transplant	10.1111/ajt.16314	USA	117	Monoclonal antibodies	32.26%
32954492	R. Yang, et al.	Eur J Clin Invest	10.1111/eci.13412	China	175	Glucocorticoids	21.43%
32960147	R. Pereda, et al.	J Interferon Cytokine Res	10.1089/jir.2020.0124	Cuba	761	Immunomodulators	21.43%
32960899	G. Ruiz-Irastorza, et al.	PLoS One	10.1371/journal.pone.0239401	Spain	242	Glucocorticoids	43.33%
32971254	M. Bartoletti, et al.	Clin Microbiol Infect	10.1016/j.cmi.2020.09.014	Italy	513	Glucocorticoids	65.52%
32979572	J. Martínez-Sanz, et al.	Clin Microbiol Infect	10.1016/j.cmi.2020.09.021	Spain	1229	Monoclonal antibodies	56.67%
32988602	O. Breik, et al.	Br J Anaesth	10.1016/j.bja.2020.08.023	UK	164	Ventilation	41.38%
32995704	F. Ionescu, et al.	TH Open	10.1055/s-0040-1716721	USA	127	Anticoagulation	53.57%
32997237	L. Ayerbe, et al.	Intern Emerg Med	10.1007/s11739-020-02505-x	UK	2075	Antimalarials	33.33%
33002613	M. A. Kaminski, et al.	Int J Infect Dis	10.1016/j.ijid.2020.09.1447	USA	125	Monoclonal antibodies	34.62%
33007454	A. J. J. Lammers, et al.	Int J Infect Dis	10.1016/j.ijid.2020.09.1460	Netherlands	1064	Antimalarials	61.54%
33007478	Z. Liu, et al.	Clin Microbiol Infect	10.1016/j.cmi.2020.09.045	China	646	Glucocorticoids	51.72%

33023669	C. Ferrando, et al.	Crit Care	10.1186/s13054-020-03314-6	Spain	199	Ventilation	57.14%
33027192	V. Russo, et al.	J Cardiovasc Pharmacol	10.1097/fjc.0000000000000893	Italy	100	Anticoagulation	26.92%
33035673	M. Rubio-Rivas, et al.	Int J Infect Dis	10.1016/j.ijid.2020.09.1486	Spain	189	Glucocorticoids	57.69%
33038227	R. Rogers, et al.	Clin Infect Dis	10.1093/cid/ciaa1548	USA	241	Other pharmaceuticals	58.62%
33043287	F. Albani, et al.	EClinicalMedicine	10.1016/j.eclinm.2020.100562	Italy	1403	Anticoagulation	68.97%
33043484	F. Ionescu, et al.	Eur J Haematol	10.1111/ejh.13533	USA	3480	Anticoagulation	61.54%
33044019	F. H. Annie, et al.	Pharmacotherapy	10.1002/phar.2467	USA	734	Antimalarials	48.28%
33047335	B. Atallah, et al.	Anaesthesia	10.1111/anae.15300	UAE	188	Anticoagulation	42.31%
33058865	S. Yeramaneni, et al.	Gastroenterology	10.1053/j.gastro.2020.10.011	USA	7000	Other pharmaceuticals	37.04%
33065103	J. C. Rajter, et al.	Chest	10.1016/j.chest.2020.10.009	USA	280	Other pharmaceuticals	58.62%
33068758	E. J. Peters, et al.	Clin Microbiol Infect	10.1016/j.cmi.2020.10.004	Netherlands	1949	Antimalarials	54.84%
33072814	F. Salton, et al.	Open Forum Infect Dis	10.1093/ofid/ofaa421	Italy	173	Glucocorticoids	67.86%
33072869	M. Rivera-Izquierdo, et al.	Med Clin (Engl Ed)	10.1016/j.medcle.2020.06.024	Spain	238	Combination of pharmaceuticals	38.46%
33075378	S. Narain, et al.	Chest	10.1016/j.chest.2020.09.275	USA	5776	Combination of pharmaceuticals	53.57%
33080002	S. Gupta, et al.	JAMA Intern Med	10.1001/jamainternmed.2020.6252	USA	3924	Monoclonal antibodies	64.52%
33090501	J. Qu, et al.	Clin Exp Pharmacol Physiol	10.1111/1440-1681.13425	China	170	Antivirals	38.46%
33093359	J. H. Chow, et al.	Anesth Analg	10.1213/ane.0000000000005292	USA	412	Anticoagulation	58.62%
33097047	W. Gao, et al.	Virol J	10.1186/s12985-020-01428-5	China	220	Antivirals	21.43%
33107607	C. Tortajada, et al.	J Med Virol	10.1002/jmv.26635	Spain	115	Glucocorticoids	57.69%
33107664	E. M. H. Padrão, et al.	Acad Emerg Med	10.1111/acem.14160	USA	166	Ventilation	82.14%
33115682	M. Górgolas Hernández-Mora, et al.	Int J Infect Dis	10.1016/j.ijid.2020.10.045	Spain	186	Monoclonal antibodies	42.31%
33117850	M. Kalligeros, et al.	Open Forum Infect Dis	10.1093/ofid/ofaa319	USA	224	Antivirals	50.00%
33122096	R. Derwand, et al.	Int J Antimicrob Agents	10.1016/j.ijantimicag.2020.106214	Germany	141	Combination of pharmaceuticals	46.15%
33127507	M. J. Choi, et al.	Int J Infect Dis	10.1016/j.ijid.2020.10.062	South Korea	4197	Combination of pharmaceuticals	58.62%
33129511	A. Rodríguez-Molinero, et al.	Med Clin (Barc)	10.1016/j.medcli.2020.08.003	Spain	187	Glucocorticoids	48.28%
33133323	T. Sandhu, et al.	Can J Infect Dis Med Microbiol	10.1155/2020/8865954	USA	112	Other pharmaceuticals	50.00%
33137493	S. N. Szente Fonseca, et al.	Travel Med Infect Dis	10.1016/j.tmaid.2020.101906	Brazil	717	Combination of pharmaceuticals	46.15%
33138975	R. Nadeem, et al.	Heart Lung	10.1016/j.hrtlng.2020.10.009	UAE	149	Anticoagulation	19.23%
33141117	J. Liu, et al.	J Clin Invest	10.1172/jci140617	China	774	Glucocorticoids	48.28%
33148005	J. Shi, et al.	Am J Chin Med	10.1142/s0192415x20500755	China	234	Traditional Chinese medicine	40.74%
33154452	A. Tsai, et al.	Sci Rep	10.1038/s41598-020-76187-y	USA	274	Monoclonal antibodies	44.44%
33165755	I. J. Núñez-Gil, et al.	Intern Emerg Med	10.1007/s11739-020-02543-5	Spain	1021	Combination of pharmaceuticals	50.00%
33172477	C. Wu, et al.	Crit Care	10.1186/s13054-020-03340-4	China	382	Glucocorticoids	72.41%
33181320	N. Shi, et al.	Pharmacol Res	10.1016/j.phrs.2020.105290	China	782	Traditional Chinese medicine	42.86%
33186991	H. H. Billett, et al.	Thromb Haemost	10.1055/s-0040-1720978	USA	3625	Anticoagulation	42.86%
33187911	C. Liu, et al.	Int Immunopharmacol	10.1016/j.intimp.2020.107157	China	473	Other pharmaceuticals	35.71%
33208294	Q. Sun, et al.	Int Immunopharmacol	10.1016/j.intimp.2020.107143	China	771	Immunomodulators	48.28%
33219551	A. Papamanoli, et al.	Eur J Clin Invest	10.1111/eci.13458	USA	447	Glucocorticoids	67.86%
33220354	G. Bozzi, et al.	J Allergy Clin Immunol	10.1016/j.jaci.2020.11.006	Italy	120	Combination of pharmaceuticals	36.67%
33225307	T. C. Lewis, et al.	Crit Care Explor	10.1097/cce.0000000000000283	USA	1000	Monoclonal antibodies	57.14%
33225952	S. Jonmarker, et al.	Crit Care	10.1186/s13054-020-03375-7	Sweden	152	Anticoagulation	57.14%
33275267	J. Y. Liu, et al.	Eur Rev Med Pharmacol Sci	10.26355/eurrev_202011_23854	China	148	Antivirals	26.92%
33277573	V. Spagnuolo, et al.	Sci Rep	10.1038/s41598-020-78039-1	Italy	280	Glucocorticoids	33.33%
33280066	B. Ruiz-Antorán, et al.	Infect Dis Ther	10.1007/s40121-020-00373-8	Spain	506	Monoclonal antibodies	61.54%

33288411	E. Cereda, et al.	Nutrition	10.1016/j.nut.2020.111055	Italy	324	Other pharmaceuticals	17.86%
33298617	J. Tian, et al.	J Immunol	10.4049/jimmunol.2000981	China	195	Monoclonal antibodies	55.56%
33307378	J. Echarte-Morales, et al.	J Electrocardiol	10.1016/j.jelectrocard.2020.11.012	Spain	168	Combination of pharmaceuticals	42.86%
33307734	K. Canoglu, et al.	Ann Saudi Med	10.5144/0256-4947.2020.462	Turkey	154	Anticoagulation	34.62%
33315350	A. Demir, et al.	Turk J Med Sci	10.3906/sag-2009-140	Turkey	109	Other pharmaceuticals	34.62%
33322317	S. F. Ling, et al.	Nutrients	10.3390/nu12123799	UK	444	Other pharmaceuticals	69.23%
33326457	R. Borie, et al.	PLoS One	10.1371/journal.pone.0243961	France	171	Combination of pharmaceuticals	50.00%
33333102	J. Tan, et al.	Virus Res	10.1016/j.virusres.2020.198262	China	333	Combination of pharmaceuticals	34.62%
33333252	M. J. Fisher, et al.	Int J Infect Dis	10.1016/j.ijid.2020.12.021	USA	115	Monoclonal antibodies	46.15%
33337933	R. Pereda, et al.	J Interferon Cytokine Res	10.1089/jir.2020.0188	Cuba	2000	Immunomodulators	23.08%
33339536	Y. Li, et al.	Crit Care	10.1186/s13054-020-03429-w	China	294	Glucocorticoids	50.00%
33342929	Y. Su, et al.	Biosci Trends	10.5582/bst.2020.03340	China	600	Antimalarials	26.92%
33357219	M. Hu, et al.	BMC Pulm Med	10.1186/s12890-020-01354-w	China	105	Ventilation	57.69%
33358502	M. Li, et al.	Am J Med Sci	10.1016/j.amjms.2020.11.005	USA	1938	Monoclonal antibodies	30.77%
33365358	M. Falcone, et al.	Open Forum Infect Dis	10.1093/ofid/ofaa563	Italy	315	Anticoagulation	48.28%
33382449	N. Vernaz, et al.	Swiss Med Wkly	10.4414/smw.2020.20446	Switzerland	840	Combination of pharmaceuticals	53.33%
33382734	X. You, et al.	PLoS One	10.1371/journal.pone.0244128	China	367	Glucocorticoids	55.56%
33382796	J. Walker, et al.	PLoS One	10.1371/journal.pone.0244857	UK	347	Ventilation	69.23%
33408529	A. Potalivo, et al.	Clin Epidemiol	10.2147/clep.S278709	Italy	1424	Ventilation	60.71%
33412395	P. Baghaei, et al.	Int Immunopharmacol	10.1016/j.intimp.2020.107329	Iran	456	Combination of pharmaceuticals	32.14%
