# Supplementary material for: Poor reporting quality of observational clinical studies comparing treatments of COVID-19 – a retrospective cross-sectional study
Source: BMC Med Res Methodol. 2022 Jan 20;22:23. doi: 10.1186/s12874-021-01501-9 (PMC8771183; doi:10.1186/s12874-021-01501-9)
Supplement: Supplementary file 4 — Additional file 4. Separate linear regression models for the percentage adherence to the STROBE checklist. The table shows the effects of the prespecified independent variables (predictors) on the percentage adherence to the STROBE checklist according to separate simple and multiple linear regression models for each predictor. [file 12874_2021_1501_MOESM4_ESM.docx]

| Additional file 4 | | | | | | | | |
| --- | --- | --- | --- | --- | --- | --- | --- | --- |
| **Independent variable** | | Point estimate of change in  percentage adherence  (Unstandardised coefficient β) | | | Standardised coefficient Beta | P-value | R^2^ | Adjusted R^2^ |
|  |  | β | 95% CI | SE |  |  |  |  |
| **Simple linear regression models for the percentage adherence to the STROBE checklist** | | | | | | | | |
| Month of publication | | .939 | -.146 to 2.024 | .549 | .144 | .089 | .021 | .014 |
| STROBE mentioned | | 9.209 | 1.832 to 16.586 | 3.731 | .206 | .015 | .042 | .035 |
| STROBE in author guidelines | | 8.537 | 4.083 to 12.991 | 2.253 | .307 | <.001 | .094 | .088 |
| **Multiple linear regression models for the percentage adherence to the STROBE checklist** | | | | | | | | |
| **Country of origin ^a^** | |  |  |  |  | .019 | .106 | .066 |
|  | China | -8.701 | -14.541 to -2.862 | 2.952 | -.293 | .004 |  | |
|  | Italy | -6.539 | -13.782 to .704 | 3.662 | -.167 | .076 |  |  |
|  | Spain | 1.742 | -6.318 to 9.801 | 4.075 | .039 | .670 |  |  |
|  | France | -2.969 | -14.293 to 8.355 | 5.725 | -.045 | .605 |  |  |
|  | Great Britain | -.258 | -12.522 to 12.005 | 6.200 | -.004 | .967 |  |  |
|  | Other countries | -9.657 | -17.033 to -2.281 | 3.729 | -.241 | .011 |  |  |
| **Impact factor ^b^** | |  |  |  |  | <.001 | .129 | .109 |
|  | 2^nd^ quartile | 8.863 | 2.848 to 14.878 | 3.042 | .286 | .004 |  | |
|  | 3^rd^ quartile | 5.515 | -.500 to 11.530 | 3.042 | .178 | .072 |  |  |
|  | 4^th^ quartile | 13.189 | 7.174 to 19.205 | 3.042 | .425 | <.001 |  |  |
| ^a^ Country estimates to be interpreted in relation to the reference country USA  ^b^ Estimates to be interpreted in relation to 1^st^ quartile. Quartile boundaries: 1^st^: 0.717 to 2.739, 2^nd^: 2.740 to 3.639, 3^rd^: 3.656 to 5.893, 4^th^: 6.407 to 74.669  Dataset n = 140; missings = 7 | | | | | | | | |

Additional file 4 –Independent linear regression models for the percentage adherence to the STROBE checklist for precpecified predictors

The table shows the effects of the prespecified independent variables (predictors) on the percentage adherence to the STROBE checklist according to separate simple and multiple linear regression models for each predictor.
