# Supplementary material for: Poor reporting quality of observational clinical studies comparing treatments of COVID-19 – a retrospective cross-sectional study
Source: BMC Med Res Methodol. 2022 Jan 20;22:23. doi: 10.1186/s12874-021-01501-9 (PMC8771183; doi:10.1186/s12874-021-01501-9)
Supplement: Supplementary file 5 — Additional file 5. Summary of excluded publications of observational studies on the treatment of COVID-19. [file 12874_2021_1501_MOESM5_ESM.pdf]

Additional file 5 – Summary of excluded publications of observational studies on the treatment of COVID-19

PDMI	Author	Journal	DOI	Reason for exclusion
32171872	Deng L et al.	J Infect	10.1016/j.jinf.2020.03.002	Number of participants
32173576	Liu F et al.	Int J Infect Dis	10.1016/j.ijid.2020.03.013	No clinical outcomes
32198776	Wan S et al.	J Med Virol	10.1002/jmv.25783	No analytical data
32220033	Wu J et al.	J Intern Med	10.1111/joim.13063	No clinical outcomes
32223012	Easom N et al.	Influenza Other Respir Viruses	10.1111/irv.12739	No clinical outcomes
32227274	Yuan J et al.	Inflamm Res	10.1007/s00011-020-01342-0	Number of participants
32236562	Huang M et al.	J Mol Cell Biol	10.1093/jmcb/mjaa014	Number of participants
32253318	Duan K et al.	Proc Natl Acad Sci U S A	10.1073/pnas.2004168117	No clinical outcomes
32253759	Luo P et al.	J Med Virol	10.1002/jmv.25801	Number of participants
32255489	Perinel S et al.	Clin Infect Dis	10.1093/cid/ciaa394	Number of participants
32266987	Zha L et al.	Med J Aust	10.5694/mja2.50577	Number of participants
32271456	Ye XT et al.	Eur Rev Med Pharmacol Sci	10.26355/eurrev_202003_20706	Number of participants
32275753	Wu Y et al.	QJM	10.1093/qjmed/hcaa121	Number of participants
32275812	Grein J et al.	N Engl J Med	10.1056/NEJMoa2007016	Number of participants
32278670	Barrasa H et al.	Anaesth Crit Care Pain Med	10.1016/j.accpm.2020.04.001	Number of participants
32283143	Zhu Z et al.	J Infect	10.1016/j.jinf.2020.03.060	Number of participants
32283325	Fan Z et al.	Clin Gastroenterol Hepatol	10.1016/j.cgh.2020.04.002	No clinical outcomes
32289548	Gautret P et al.	Travel Med Infect Dis	10.1016/j.tmaid.2020.101663	Number of participants
32320677	Giamarellos-Bourboulis EJ et al.	Cell Host Microbe	10.1016/j.chom.2020.04.009	No clinical outcomes
32321905	Sato K et al.	Biosci Trends	10.5582/bst.2020.03082	No clinical outcomes
32324898	Sun J et al.	Clin Pharmacol Ther	10.1002/cpt.1866	No clinical outcomes
32329231	Testa S et al.	J Thromb Haemost	10.1111/jth.14871	No clinical outcomes
32330343	Pereira MR et al.	Am J Transplant	10.1111/ajt.15941	No clinical outcomes
32344167	Lian N et al.	Clin Microbiol Infect	10.1016/j.cmi.2020.04.026	Number of participants
32347743	Saleh M et al.	Circ Arrhythm Electrophysiol	10.1161/CIRCEP.120.008662	No clinical outcomes
32348485	Zeng QL et al.	J Infect Dis	10.1093/infdis/jiaa228	Number of participants
32350134	Xu X et al.	Proc Natl Acad Sci U S A	10.1073/pnas.2005615117	Number of participants
32353223	Zangrillo A et al.	Crit Care Resusc	missing	No clinical outcomes
32369191	Alattar R et al.	J Med Virol	10.1002/jmv.25964	Number of participants
32387320	Klopfenstein T et al.	Med Mal Infect	10.1016/j.medmal.2020.05.001	Number of participants
32387409	Million M et al.	Travel Med Infect Dis	10.1016/j.tmaid.2020.101738	No analytical data
32403946	Stroppa EM et al.	Future Oncol	10.2217/fon-2020-0369	No clinical outcomes
32404630	Prabhakaran K et al.	J Trauma Acute Care Surg	10.1097/TA.0000000000002780	No clinical outcomes
32405160	Capra R et al.	Eur J Intern Med	10.1016/j.ejim.2020.05.009	Number of participants
32407884	Chorin E et al.	Heart Rhythm	10.1016/j.hrthm.2020.05.014	No clinical outcomes
32409150	Vouri SM et al.	Res Social Adm Pharm	10.1016/j.sapharm.2020.04.031	No clinical outcomes
32410206	Huang Q et al.	Int J Clin Pharm	10.1007/s11096-020-01031-2	Number of participants
32410714	Yu Y et al.	Crit Care	10.1186/s13054-020-02939-x	No analytical data
32411770	Jiang Y et al.	Ann Transl Med	10.21037/atm.2020.04.20	No clinical outcomes
32418730	Gérard A et al.	Therapie	10.1016/j.therap.2020.05.002	No clinical outcomes
32420751	Zhang HT et al.	Am J Chin Med	10.1142/S0192415X20500391	Number of participants
32423903	Stoneham SM et al.	Clin Med (Lond)	10.7861/clinmed.2020-0228	No analytical data
32427613	Yang X et al.	Crit Care Med	10.1097/CCM.0000000000004447	Number of participants
32437770	Goicoechea M et al.	Kidney Int	10.1016/j.kint.2020.04.031	No clinical outcomes

32439366	Cipriani A et al.	Int J Cardiol	10.1016/j.ijcard.2020.05.036	No clinical outcomes
32441771	Ross SB et al.	J Am Geriatr Soc	10.1111/jgs.16623	No analytical data
32441786	Gong Y et al.	J Med Virol	10.1002/jmv.26052	Number of participants
32446167	Peng H et al.	J Clin Virol	10.1016/j.jcv.2020.104425	No clinical outcomes
32446698	Freedberg DE et al.	Gastroenterology	10.1053/j.gastro.2020.05.053	Number of participants
32447102	Marfella R et al.	Diabetes Metab	10.1016/j.diabet.2020.05.005	No clinical outcomes
32449128	Rogado J et al.	Clin Transl Oncol	10.1007/s12094-020-02381-z	No analytical data
32463348	Ramireddy A et al.	J Am Heart Assoc	10.1161/JAHA.120.017144	No clinical outcomes
32473109	Salazar E et al.	Am J Pathol	10.1016/j.ajpath.2020.05.014	No analytical data
32473657	Vizcarra P et al.	Lancet HIV	10.1016/S2352-3018(20)30164-8	No clinical outcomes
32473681	Kuderer NM et al.	Lancet	10.1016/S0140-6736(20)31187-9	No clinical outcomes
32475019	Irie K et al.	Clin Transl Sci	10.1111/cts.12827	No clinical outcomes
32482597	Campochiaro C et al.	Eur J Intern Med	10.1016/j.ejim.2020.05.021	Number of participants
32492211	Zuo Y et al.	J Med Virol	10.1002/jmv.26127	No clinical outcomes
32495917	Ding JG et al.	Eur Rev Med Pharmacol Sci	10.26355/eurrev_202005_21373	No analytical data
32501538	Zhang Z et al.	J Med Virol	10.1002/jmv.26141	Number of participants
32505076	Rogado J et al.	Lung Cancer	10.1016/j.lungcan.2020.05.034	No analytical data
32506862	Liu Z et al.	J Tradit Chin Med	10.19852/j.cnki.jtcm.2020.03.016	Number of participants
32510169	Chen W et al.	J Med Virol	10.1002/jmv.26142	Number of participants
32515499	Quartuccio L et al.	J Med Virol	10.1002/jmv.26149	No clinical outcomes
32518419	La Rosée F et al.	Leukemia	10.1038/s41375-020-0891-0	No clinical outcomes
32525844	Joyner MJ et al.	J Clin Invest	10.1172/JCI140200	No clinical outcomes
32531138	Zeng QL et al.	Transbound Emerg Dis	10.1111/tbed.13674	No clinical outcomes
32535147	Rinott E et al.	Clin Microbiol Infect	10.1016/j.cmi.2020.06.003	No clinical outcomes
32536150	Kim JW et al.	Korean J Intern Med	10.3904/kjim.2020.224	Number of participants
32549293	Singh AP et al.	J Clin Med	10.3390/jcm9061867	No clinical outcomes
32556143	Paccoud O et al.	Clin Infect Dis	10.1093/cid/ciaa791	Number of participants
32562159	Vahedi E et al.	Daru	10.1007/s40199-020-00353-w	Number of participants
32564984	Callejas Rubio JL et al.	Rev Esp Geriatr Gerontol	10.1016/j.regg.2020.05.004	No clinical outcomes
32569585	Coppo A et al.	Lancet Respir Med	10.1016/S2213-2600(20)30268-X	Number of participants
32570995	Selvaraj V et al.	R I Med J (2013)	missing	Number of participants
32573419	Conrozier T et al.	Clin Exp Rheumatol	missing	No analytical data
32574262	Zhou Q et al.	Front Immunol	10.3389/fimmu.2020.01061	Number of participants
32574789	Lohse A et al.	Microbes Infect	10.1016/j.micinf.2020.06.005	Number of participants
32575124	Jordan SC et al.	Clin Infect Dis	10.1093/cid/ciaa812	No analytical data
32585284	Khamis F et al.	Int J Infect Dis	10.1016/j.ijid.2020.06.064	Number of participants
32588427	Bun SS et al.	Clin Pharmacol Ther	10.1002/cpt.1968	No clinical outcomes
32589775	Humeniuk R et al.	Clin Transl Sci	10.1111/cts.12840	No clinical outcomes
32597466	Titanji BK et al.	Clin Infect Dis	10.1093/cid/ciaa879	Number of participants
32602262	Navarro-Millán I et al.	Arthritis Rheumatol	10.1002/art.41422	No clinical outcomes
32610165	Hooks M et al.	Heart Rhythm	10.1016/j.hrthm.2020.06.029	No clinical outcomes
32614258	McGuinness G et al.	Radiology	10.1148/radiol.2020202352	No clinical outcomes
32617986	Salacup G et al.	J Med Virol	10.1002/jmv.26252	No analytical data
32618699	Trigonis RA et al.	Crit Care Med	10.1097/CCM.0000000000004472	No clinical outcomes
32618700	Parzy G et al.	Crit Care Med	10.1097/CCM.0000000000004504	No clinical outcomes
32619760	Chen X et al.	Int J Infect Dis	10.1016/j.ijid.2020.06.091	No clinical outcomes
32620597	Della-Torre E et al.	Ann Rheum Dis	10.1136/annrheumdis-2020-218122	Number of participants
32621881	Samuel S et al.	Heart Rhythm	10.1016/j.hrthm.2020.06.033	No clinical outcomes
32628003	Knorr JP et al.	J Med Virol	10.1002/jmv.26191	Number of participants
32628040	Mei F et al.	Arterioscler Thromb Vasc Biol	10.1161/ATVBAHA.120.314779	No clinical outcomes

32629085	Yamasaki Y et al.	Virus Res	10.1016/j.virusres.2020.198089	Number of participants
32633860	Parker JE et al.	Mov Disord	10.1002/mds.28198	No clinical outcomes
32639062	Gatti M et al.	Br J Clin Pharmacol	10.1111/bcp.14459	No clinical outcomes
32639466	Bhumbra S et al.	Pediatr Crit Care Med	10.1097/PCC.0000000000002511	No analytical data
32641296	Marzolini C et al.	Antimicrob Agents Chemother	10.1128/AAC.01177-20	No clinical outcomes
32644223	Li TZ et al.	J Med Virol	10.1002/jmv.26280	Number of participants
32644254	Antony SJ et al.	J Med Virol	10.1002/jmv.26288	Number of participants
32646770	Morrison AR et al.	J Autoimmun	10.1016/j.jaut.2020.102512	Number of participants
32652521	Bisogno G et al.	J Pediatric Infect Dis Soc	10.1093/jpids/piaa088	No analytical data
32653015	Lecronier M et al.	Crit Care	10.1186/s13054-020-03117-9	Number of participants
32654098	Hsia BC et al.	J Interv Card Electrophysiol	10.1007/s10840-020-00822-x	No clinical outcomes
32654422	Pérez-Sáez MJ et al.	Am J Transplant	10.1111/ajt.16192	Number of participants
32662690	Hiedra R et al.	Expert Rev Anti Infect Ther	10.1080/14787210.2020.1794819	Number of participants
32672860	Fernández-Ruiz M et al.	J Med Virol	10.1002/jmv.26308	Number of participants
32677113	Simmering JE et al.	Pharmacotherapy	10.1002/phar.2445	No clinical outcomes
32686633	Tuncer T et al.	Cardiol Young	10.1017/S1047951120002425	No clinical outcomes
32688374	Lê MP et al.	J Antimicrob Chemother	10.1093/jac/dkaa261	No clinical outcomes
32689588	Dogra S et al.	J Stroke Cerebrovasc Dis	10.1016/j.jstrokecerebrovasdis.2020.104984	No analytical data
32690352	Moreno-Pérez O et al.	J Autoimmun	10.1016/j.jaut.2020.102523	No clinical outcomes
32692462	Batteux B et al.	Br J Clin Pharmacol	10.1111/bcp.14489	Number of participants
32692874	Pesavento R et al.	J Thromb Haemost	10.1111/jth.15022	No clinical outcomes
32693646	Peng L et al.	Expert Opin Drug Saf	10.1080/14740338.2020.1799975	No clinical outcomes
32693650	Tomasiewicz K et al.	Expert Rev Anti Infect Ther	10.1080/14787210.2020.1800453	Number of participants
32699149	Cauchois R et al.	Proc Natl Acad Sci U S A	10.1073/pnas.2009017117	Number of participants
32701969	Satlin MJ et al.	PLoS One	10.1371/journal.pone.0236778	No clinical outcomes
32703883	Vianello A et al.	Thorax	10.1136/thoraxjnl-2020-214993	Number of participants
32709280	Huang M et al.	Am J Med Sci	10.1016/j.amjms.2020.05.038	No clinical outcomes
32718719	Brandariz-Nuñez D et al.	Med Clin (Barc)	10.1016/j.medcli.2020.06.026	No clinical outcomes
32719218	Patel A et al.	Indian J Med Microbiol	10.4103/ijmm.IJMM_20_298	Number of participants
32719218	Patel A et al.	Indian J Med Microbiol	10.4103/ijmm.IJMM_20_298	No analytical data
32720612	Mareev VY et al.	Kardiologiia	10.18087/cardio.2020.6.n1226	No clinical outcomes
32721580	Montastruc F et al.	Clin Gastroenterol Hepatol	10.1016/j.cgh.2020.07.050	No clinical outcomes
32726724	Keske Ş et al.	Int J Infect Dis	10.1016/j.ijid.2020.07.036	Number of participants
32750201	Hu F et al.	Clin Respir J	10.1111/crj.13243	No clinical outcomes
32753141	Voisin O et al.	Mayo Clin Proc	10.1016/j.mayocp.2020.05.005	No clinical outcomes
32758308	Glibbery N et al.	J Laryngol Otol	10.1017/S0022215120001759	Number of participants
32766535	Mastroianni A et al.	EClinicalMedicine	10.1016/j.eclinm.2020.100410	Number of participants
32767349	Ma Q et al.	Eur Rev Med Pharmacol Sci	10.26355/eurrev_202008_22508	Number of participants
32768693	Langer-Gould A et al.	Int J Infect Dis	10.1016/j.ijid.2020.07.081	Number of participants
32768701	Potere N et al.	Int J Infect Dis	10.1016/j.ijid.2020.07.078	Number of participants
32768975	Xin S et al.	Biomed Pharmacother	10.1016/j.biopha.2020.110500	Number of participants
32773165	Rivera-Izquierdo M et al.	Med Clin (Barc)	10.1016/j.medcli.2020.06.025	No clinical outcomes
32777263	Lopez A et al.	Int J Antimicrob Agents	10.1016/j.ijantimicag.2020.106136	Number of participants
32779755	Xue H et al.	J Med Virol	10.1002/jmv.26193	No analytical data
32784217	Montesarchio V et al.	J Immunother Cancer	10.1136/jitc-2020-001089	Number of participants
32789513	van de Veerdonk FL et al.	JAMA Netw Open	10.1001/jamanetworkopen.2020.17708	Number of participants
32790075	Pettit NN et al.	J Med Virol	10.1002/jmv.26429	No clinical outcomes
32792492	Liu F et al.	Sci Rep	10.1038/s41598-020-70387-2	No analytical data
32795898	Franzini M et al.	Int Immunopharmacol	10.1016/j.intimp.2020.106879	No analytical data
32808695	Goncalves Mendes Neto A et al.	J Med Virol	10.1002/jmv.26441	No clinical outcomes

32809969	Bronte V et al.	J Clin Invest	10.1172/JCI141772	No clinical outcomes
32815344	Gundem T et al.	Tidsskr Nor Laegeforen	10.4045/tidsskr.20.0445	No clinical outcomes
32820807	Vaughn VM et al.	Clin Infect Dis	10.1093/cid/ciaa1239	No clinical outcomes
32826818	Zhou S et al.	Shock	10.1097/SHK.0000000000001629	No analytical data
32827987	Bakhshaliyev N et al.	J Electrocardiol	10.1016/j.jelectrocard.2020.08.008	No clinical outcomes
32828896	Dubernet A et al.	J Glob Antimicrob Resist	10.1016/j.jgar.2020.08.001	Number of participants
32829390	Pasquini Z et al.	J Antimicrob Chemother	10.1093/jac/dkaa321	Number of participants
32843231	Iglesias-Julián E et al.	J Autoimmun	10.1016/j.jaut.2020.102537	Number of participants
32844224	Giaime P et al.	Nephrol Dial Transplant	10.1093/ndt/gfaa191	No clinical outcomes
32853675	Padilla S et al.	Int J Antimicrob Agents	10.1016/j.ijantimicag.2020.106142	No clinical outcomes
32855187	Masur J et al.	AJNR Am J Neuroradiol	10.3174/ajnr.A6728	Number of participants
32856202	Cag Y et al.	Eur J Clin Microbiol Infect Dis	10.1007/s10096-020-04016-1	No analytical data
32856282	Cojutti PG et al.	Clin Pharmacokinet	10.1007/s40262-020-00933-8	No clinical outcomes
32858566	Levy C et al.	Am J Gastroenterol	10.14309/ajg.0000000000000828	No clinical outcomes
32860432	Okoh AK et al.	J Med Virol	10.1002/jmv.26471	Number of participants
32861333	Joyner MJ et al.	Mayo Clin Proc	10.1016/j.mayocp.2020.06.028	No analytical data
32871242	Hogan Ii RB et al.	Pulm Pharmacol Ther	10.1016/j.pupt.2020.101942	No analytical data
32881340	Shi C et al.	Clin Transl Sci	10.1111/cts.12880	Number of participants
32889236	Zain Mushtaq M et al.	Int Immunopharmacol	10.1016/j.intimp.2020.106926	No analytical data
32890069	Inama G et al.	J Cardiovasc Med (Hagerstown)	10.2459/JCM.0000000000001066	No analytical data
32898149	Lund LC et al.	PLoS Med	10.1371/journal.pmed.1003308	No clinical outcomes
32900781	Kirenga B et al.	BMJ Open Respir Res	10.1136/bmjresp-2020-000646	No clinical outcomes
32907357	Friedman DM et al.	Circ Arrhythm Electrophysiol	10.1161/CIRCEP.120.008686	No clinical outcomes
32907890	Mouhat B et al.	Eur Respir J	10.1183/13993003.01811-2020	No clinical outcomes
32912404	Li T et al.	Zhonghua Wei Zhong Bing Ji Jiu Yi Xue	10.3760/cma.j.cn121430-20200513-00656	No clinical outcomes
32912961	Albertini L et al.	Eur J Hosp Pharm	10.1136/ejhpharm-2020-002414	Number of participants
32916565	Musoke N et al.	Thromb Res	10.1016/j.thromres.2020.08.035	No clinical outcomes
32920503	Stattin K et al.	J Crit Care	10.1016/j.jcrc.2020.08.026	Number of participants
32920570	Ayaz CM et al.	Infez Med	missing	No clinical outcomes
32920751	Osawa I et al.	J Thromb Thrombolysis	10.1007/s11239-020-02275-5	No analytical data
32925581	Zhang Q et al.	J Trauma Acute Care Surg	10.1097/TA.0000000000002939	Number of participants
32926977	Yousaf A et al.	J Am Acad Dermatol	10.1016/j.jaad.2020.09.009	No clinical outcomes
32927129	Tan Q et al.	Bioorg Chem	10.1016/j.bioorg.2020.104257	Number of participants
32932329	Kanburoglu MK et al.	Pediatr Infect Dis J	10.1097/INF.0000000000002862	No clinical outcomes
32934372	Liu STH et al.	Nat Med	10.1038/s41591-020-1088-9	Number of participants
32936252	Mercuro NJ et al.	JAMA Cardiol	10.1001/jamacardio.2020.1834	No clinical outcomes
32936266	Bessière F et al.	JAMA Cardiol	10.1001/jamacardio.2020.1787	No clinical outcomes
32938838	Kishaba T et al.	Tohoku J Exp Med	10.1620/tjem.252.103	No clinical outcomes
32943233	Rambaldi A et al.	Immunobiology	10.1016/j.imbio.2020.152001	Number of participants
32950003	Masiá M et al.	EBioMedicine	10.1016/j.ebiom.2020.102999	No clinical outcomes
32954754	Guo H et al.	Ann Palliat Med	10.21037/apm-20-1478	Number of participants
32959052	Hueso T et al.	Blood	10.1182/blood.2020008423	Number of participants
32959400	Liu W et al.	J Intern Med	10.1111/joim.13160	No clinical outcomes
32959730	Udi J et al.	J Intensive Care Med	10.1177/0885066620954364	Number of participants
32965359	Yaylaci S et al.	Rev Assoc Med Bras (1992)	10.1590/1806-9282.66.S2.65	No clinical outcomes
32965715	Omrani AS et al.	J Med Virol	10.1002/jmv.26537	Number of participants
32977129	Pavoni V et al.	Thromb Res	10.1016/j.thromres.2020.09.013	Number of participants
32984833	Barkama R et al.	Crit Care Explor	10.1097/CCE.0000000000000207	Number of participants
32993751	Menzella F et al.	Crit Care	10.1186/s13054-020-03306-6	Number of participants
33003194	Jing Y et al.	Brief Bioinform	10.1093/bib/bbaa234	No clinical outcomes

33003964	Saleh M et al.	Circ Arrhythm Electrophysiol	10.1161/CIRCEP.120.008937	No clinical outcomes
33008327	Wang B et al.	BMC Infect Dis	10.1186/s12879-020-05425-5	Number of participants
33009770	Nachega JB et al.	Am J Trop Med Hyg	10.4269/ajtmh.20-1240	No clinical outcomes
33015160	Fu HY et al.	Biomed Res Int	10.1155/2020/2854186	No clinical outcomes
33029193	Shan W et al.	Comput Math Methods Med	10.1155/2020/1391583	No clinical outcomes
33031081	Li M et al.	J Infect Dev Ctries	10.3855/jidc.13491	Number of participants
33031085	Meneses Calderón J et al.	J Infect Dev Ctries	10.3855/jidc.13274	No analytical data
33031409	Correale P et al.	PLoS One	10.1371/journal.pone.0239692	Number of participants
33033405	Guillén L et al.	Sci Rep	10.1038/s41598-020-74001-3	Number of participants
33039952	Tan CW et al.	Nutrition	10.1016/j.nut.2020.111017	Number of participants
33040252	Aranda-Abreu GE et al.	Pharmacol Rep	10.1007/s43440-020-00168-1	Number of participants
33043651	Omidi N et al.	J Card Surg	10.1111/jocs.14707	No analytical data
33048786	Vecchio G et al.	Medicina (B Aires)	missing	No clinical outcomes
33051534	Holt GE et al.	Sci Rep	10.1038/s41598-020-74328-x	Number of participants
33054630	Li W et al.	Ther Adv Respir Dis	10.1177/1753466620963017	No clinical outcomes
33055313	Giampreti A et al.	J Perinat Med	10.1515/jpm-2020-0339	No clinical outcomes
33063035	Sirimaturos M et al.	Crit Care Explor	10.1097/CCE.0000000000000232	Number of participants
33064694	Sosa-García JO et al.	Cir Cir	10.24875/CIRU.20000675	No clinical outcomes
33065275	Annweiler C et al.	J Steroid Biochem Mol Biol	10.1016/j.jsbmb.2020.105771	Number of participants
33066885	Carneiro T et al.	J Stroke Cerebrovasc Dis	10.1016/j.jstrokecerebrovasdis.2020.105201	No clinical outcomes
33069626	Zhang X et al.	J Integr Med	10.1016/j.joim.2020.10.002	Number of participants
33078076	Gamberini L et al.	J Intensive Care	10.1186/s40560-020-00499-4	No clinical outcomes
33078372	Garcia P et al.	Drug Saf	10.1007/s40264-020-01013-3	No clinical outcomes
33093279	Caplan M et al.	Crit Care Med	10.1097/CCM.0000000000004711	No clinical outcomes
33111169	Faíco-Filho KS et al.	Braz J Microbiol	10.1007/s42770-020-00395-x	Number of participants
33128800	Søvik S et al.	Acta Anaesthesiol Scand	10.1111/aas.13726	Number of participants
33129099	Valizadeh H et al.	Int Immunopharmacol	10.1016/j.intimp.2020.107088	Number of participants
33136164	Lee S et al.	J Am Osteopath Assoc	10.7556/jaoa.2020.156	Number of participants
33136589	Guihaire J et al.	ASAIO J	10.1097/MAT.0000000000001251	No clinical outcomes
33138974	Ñamendys-Silva SA et al.	Heart Lung	10.1016/j.hrtlng.2020.10.013	No clinical outcomes
33150470	Cattaneo D et al.	Drugs Aging	10.1007/s40266-020-00812-8	No clinical outcomes
33151482	Lin KJ et al.	Drugs	10.1007/s40265-020-01424-7	No clinical outcomes
33153629	Temesgen Z et al.	Mayo Clin Proc	10.1016/j.mayocp.2020.08.038	Number of participants
33154498	Cohen IV et al.	Sci Rep	10.1038/s41598-020-76258-0	No clinical outcomes
33160408	Rizzi S et al.	BMC Res Notes	10.1186/s13104-020-05358-x	No clinical outcomes
33160854	Li X et al.	Int Immunopharmacol	10.1016/j.intimp.2020.107022	No clinical outcomes
33161002	Salvati L et al.	Immunol Lett	10.1016/j.imlet.2020.10.009	Number of participants
33164544	Gunay S et al.	Bratisl Lek Listy	10.4149/BLL_2020_134	No clinical outcomes
33165762	de Cáceres C et al.	Pharmacol Rep	10.1007/s43440-020-00186-z	Number of participants
33168413	Liu C et al.	Int Immunopharmacol	10.1016/j.intimp.2020.107121	No clinical outcomes
33175880	Camprubí D et al.	PLoS One	10.1371/journal.pone.0242184	No analytical data
33180360	Mo Y et al.	J Clin Pharmacol	10.1002/jcph.1787	Number of participants
33181454	Çap M et al.	J Electrocardiol	10.1016/j.jelectrocard.2020.10.015	No clinical outcomes
33181718	Wang Y et al.	Medicine (Baltimore)	10.1097/MD.0000000000023257	No clinical outcomes
33186704	Patterson BK et al.	Int J Infect Dis	10.1016/j.ijid.2020.10.101	No analytical data
33187475	Hong L et al.	BMC Infect Dis	10.1186/s12879-020-05528-z	No analytical data
33188624	Roumier M et al.	J Clin Immunol	10.1007/s10875-020-00911-6	Number of participants
33189888	Corominas H et al.	Clin Immunol	10.1016/j.clim.2020.108631	No analytical data
33196991	Bolzetta F et al.	Aging Clin Exp Res	10.1007/s40520-020-01750-6	Number of participants
33198670	Arnold F et al.	BMC Nephrol	10.1186/s12882-020-02150-8	Number of participants

33200828	Hill JA et al.	J Med Virol	10.1002/jmv.26674	Number of participants
33204428	Ji F et al.	New Microbes New Infect	10.1016/j.nmni.2020.100814	No analytical data
33219294	Lim JH et al.	Sci Rep	10.1038/s41598-020-76915-4	No clinical outcomes
33231925	Abbas HM et al.	Int J Clin Pract	10.1111/ijcp.13856	No clinical outcomes
33232277	Han D et al.	Aging (Albany NY)	10.18632/aging.202172	No clinical outcomes
33234007	Sonti R et al.	J Intensive Care Med	10.1177/0885066620976525	Number of participants
33234138	Seeland U et al.	BMC Med	10.1186/s12916-020-01851-z	No clinical outcomes
33236646	Esen F et al.	Curr Med Res Opin	10.1080/03007995.2020.1856058	Number of participants
33237291	Hubiche T et al.	JAMA Dermatol	10.1001/jamadermatol.2020.4324	No clinical outcomes
33249281	Tsai KC et al.	Biomed Pharmacother	10.1016/j.biopha.2020.111037	Number of participants
33256382	Cesare Perotti et al.	Haematologica	10.3324/haematol.2020.261784	No analytical data
33262433	Macías J et al.	Sci Rep	10.1038/s41598-020-78029-3	No clinical outcomes
33264556	WHO Solidarity Trial Consortium	N Engl J Med	10.1056/NEJMoa2023184	No clinical outcomes
33287864	Perier F et al.	Crit Care	10.1186/s13054-020-03414-3	Number of participants
33290446	Vaira LA et al.	Rhinology	10.4193/Rhin20.515	Number of participants
33291194	Seyhan AU et al.	J Coll Physicians Surg Pak	10.29271/jcpsp.2020.supp2.S153	No clinical outcomes
33292350	Saavedra D et al.	Immun Ageing	10.1186/s12979-020-00207-8	Number of participants
33302991	Kooistra EJ et al.	Crit Care	10.1186/s13054-020-03364-w	Number of participants
33305475	Del Borrello G et al.	J Thromb Haemost	10.1111/jth.15216	Number of participants
33307658	Bodrova RA et al.	Vopr Kurortol Fizioter Lech Fiz Kult	10.17116/kurort20209706111	Number of participants
33309764	Denas G et al.	Int J Cardiol	10.1016/j.ijcard.2020.12.024	No clinical outcomes
33310665	Hernández A et al.	Int Immunopharmacol	10.1016/j.intimp.2020.107261	Number of participants
33311551	Landi L et al.	Sci Rep	10.1038/s41598-020-78492-y	Number of participants
33316806	Herth FJF et al.	Respiration	10.1159/000511376	Number of participants
33318314	Shi J et al.	Aging (Albany NY)	10.18632/aging.202223	Number of participants
33319213	Weiss KD et al.	JTCVS Tech	10.1016/j.xjtc.2020.11.016	Number of participants
33330582	Yue X et al.	Front Nutr	10.3389/fnut.2020.581679	Number of participants
33333393	Widysanto A et al.	Cytokine	10.1016/j.cyto.2020.155393	Number of participants
33336766	Bocci MG et al.	Eur Rev Med Pharmacol Sci	10.26355/eurrev_202012_24043	Number of participants
33350752	Mallat J et al.	Medicine (Baltimore)	10.1097/MD.0000000000023720	Number of participants
33353546	Vu CA et al.	BMC Infect Dis	10.1186/s12879-020-05701-4	Number of participants
33354635	Aiswarya D et al.	Kidney Int Rep	10.1016/j.ekir.2020.12.003	Number of participants
33354678	Li J et al.	Crit Care Explor	10.1097/CCE.0000000000000307	Number of participants
33355217	Luo P et al.	Clin Med (Lond)	10.7861/clinmed.2020-0348	Number of participants
33380371	Pascual Pareja JF et al.	Med Clin (Barc)	10.1016/j.medcli.2020.11.004	No clinical outcomes
33388006	Procter BC et al.	Rev Cardiovasc Med	10.31083/j.rcm.2020.04.260	No analytical data
33393643	Niwas R et al.	Adv Respir Med	10.5603/ARM.a2020.0139	Number of participants
33402859	Saggi SJ et al.	Clin Med Insights Circ Respir Pulm Med	10.1177/1179548420980699	Number of participants
33425957	Siempos II et al.	Front Med (Lausanne)	10.3389/fmed.2020.614152	Number of participants
33541491	Gao X et al.	Zhonghua Wei Zhong Bing Ji Jiu Yi Xue	10.3760/cma.j.cn121430-20201019-00679	Number of participants
